# Regulatory Elements Inserted into AAVs Confer Preferential Activity in Cortical Interneurons

**DOI:** 10.1523/ENEURO.0211-20.2020

**Published:** 2020-12-10

**Authors:** Anna N. Rubin, Ruchi Malik, Kathleen K. A. Cho, Kenneth J. Lim, Susan Lindtner, Sarah E. Robinson Schwartz, Daniel Vogt, Vikaas S. Sohal, John L. R. Rubenstein

**Affiliations:** 1 Nina Ireland Laboratory of Developmental Neurobiology, Department of Psychiatry, University of California San Francisco Weill Institute for Neurosciences, University of California San Francisco, San Francisco, CA 94158; 2 Center for Integrative Neuroscience, Department of Psychiatry, University of California San Francisco Weill Institute for Neurosciences, Kavli Institute for Fundamental Neuroscience, University of California San Francisco, San Francisco, CA 94158

**Keywords:** AAV, cortical interneurons, enhancers, fast spiking, regular spiking

## Abstract

Cortical interneuron (CIN) dysfunction is thought to play a major role in neuropsychiatric conditions like epilepsy, schizophrenia and autism. It is therefore essential to understand how the development, physiology, and functions of CINs influence cortical circuit activity and behavior in model organisms such as mice and primates. While transgenic driver lines are powerful tools for studying CINs in mice, this technology is limited in other species. An alternative approach is to use viral vectors such as AAV, which can be used in multiple species including primates and also have potential for therapeutic use in humans. Thus, we sought to discover gene regulatory enhancer elements (REs) that can be used in viral vectors to drive expression in specific cell types. The present study describes the systematic genome-wide identification of putative REs (pREs) that are preferentially active in immature CINs by histone modification chromatin immunoprecipitation and sequencing (ChIP-seq). We evaluated two novel pREs in AAV vectors, alongside the well-established *Dlx I12b* enhancer, and found that they drove CIN-specific reporter expression in adult mice. We also showed that the identified *Arl4d* pRE could drive sufficient expression of channelrhodopsin for optogenetic rescue of behavioral deficits in the *Dlx5/6*^+/−^ mouse model of fast-spiking CIN dysfunction.

## Significance Statement

Uncovering the contribution of cortical interneuron (CIN) dysfunction to neuropsychiatric conditions is pivotal to generating therapies for these debilitating disorders. To this end, it is important to study the development, physiology, and function of CINs in model organisms. While transgenic driver lines are powerful tools to study CINs in mice, this technology is limited in other species. As an alternative, gene regulatory enhancer elements (REs) can be used in viral vectors to drive expression in multiple species including primates. The present study describes the genome-wide identification of REs preferentially active in CINs and the use of these RE AAVs for CIN-specific targeting in adult mice. The methodology and novel REs described here provide new tools for studying and targeting CINs.

## Introduction

GABAergic cortical interneurons (CINs) constitute a diverse population of biochemically, physiologically and morphologically distinct neuronal subtypes that have specialized functions within cortical circuitry (Tremblay et al., 2016; Fishell and Kepecs, 2020). Based on their neurochemical and cellular properties, CINs can be broadly divided into three major subtypes: (1) parvalbumin (PV)-expressing CINs, which have fast-spiking physiological properties and innervate the perisomatic or axonal regions of excitatory pyramidal neurons (PNs); (2) somatostatin (SST)-expressing CINs, which typically have regular-spiking properties and target distal dendritic regions of PNs; and (3) vasoactive peptide (VIP)-expressing CINs, which have irregular-spiking properties and cause disinhibition of PNs by preferentially targeting other CINs. Multiple lines of evidence highlight that these CIN subtypes play dissociable roles in cortical circuits. In particular, fast-spiking PV^+^ CINs promote cortical γ oscillations, which are necessary for cognitive functions such as attention, working memory, and cognitive flexibility (Cardin et al., 2009; Sohal et al., 2009; Cho et al., 2015), and SST^+^ CINs play important roles in cortical β oscillations, input integration, and sensory processing (Yavorska and Wehr, 2016; Chen et al., 2017).

Our understanding of the development and functional heterogeneity of CINs owes heavily to the use of transgenic mouse lines expressing Cre recombinase in molecularly specified CIN subtypes (Taniguchi et al., 2011). Although these Cre-driver lines have been extremely valuable to the field, the current reliance on them has some limitations. First, while Cre-driver lines can be used in combination with other recombinases (such as Flp) to label different subtypes of neurons, the ability to manipulate and record from multiple classes of CINs simultaneously or study interactions of CINs with specific types of PNs has been lacking. Second, the Cre-driver lines are not able to distinguish between additional subclasses within the PV, SST, and VIP CIN subtypes, which have distinct physiological properties and molecular markers (Nigro et al., 2018). Third, while Cre-driver lines are widely available for mice, this technology is currently unsuitable for research in other species such as non-human primates.

A large number of studies implicate loss or alteration of the activity of CIN subtypes, particularly PV^+^ fast-spiking CINs, in neurologic diseases such as schizophrenia, epilepsy and autism (Lewis et al., 2012; Cho and Sohal, 2014; Vogt et al., 2015, 2017; Jiang et al., 2016; Hashemi et al., 2017; Malik et al., 2019). To develop targeted treatments for these disorders, it is imperative to develop technologies that will allow manipulation of the activity of CIN subtypes in humans.

The use of recombinant AAV vectors has become a widespread technique for labeling neurons as well as monitoring and modifying their electrophysiological activity (Betley and Sternson, 2011). For instance, AAV1, AAV5, and AAV9 disperse throughout the brain readily and can infect most neurons (Burger et al., 2004; Taymans et al., 2007; Nathanson et al., 2009b; Watakabe et al., 2015). AAVs have the added benefit of being translatable across species and are becoming a promising avenue for human therapeutic intervention (Haggerty et al., 2020).

Despite these advantages, limited viral genome capacity has hampered the generation of AAVs to target specific CIN subclasses. Because of their small size (often <1 kb) and specific patterns of activity, gene regulatory enhancer elements (REs) such as enhancers have proven to be a good tool for driving CIN-specific expression by viral methods. In particular, *Dlx1/2* RE *I12b*, which labels CINs in transgenic mice (Potter et al., 2009), has been used in lentiviral (Arguello et al., 2013; Vogt et al., 2014) and AAV vectors to drive the expression of fluorophores and channelrhodopsin (Lee et al., 2014a,b; Cho et al., 2015). Subsequent work showed that another RE located at the *Dlx5/6* locus has similar properties and demonstrated additional applications of this approach (Dimidschstein et al., 2016). However, viral tools targeting subclasses of CINs are in their infancy, and many still require the use of intersectional recombinase-based strategies (Mehta et al., 2019).

Here, we describe a systematic genome-wide identification of REs putatively active in immature CINs. From these, we selected two candidates and tested their utility in AAV vectors. We found that these RE AAVs drove robust, CIN-specific expression when injected into the medial prefrontal cortex (mPFC). Notably, we observed that the RE AAVs differentially target distinct CIN populations; specifically, they have a different preference for CINs with fast-spiking versus regular-spiking properties. We also demonstrate that the AAV with higher specificity for fast-spiking CINs can drive sufficient expression for *in vivo* optogenetic rescue of cognitive flexibility deficits in *Dlx5/6*^+/−^ heterozygous mutant mice.

## Materials and Methods

### Mice

All animal care, procedures, and experiments were conducted in accordance with the NIH guidelines and approved by the University of California San Francisco animal care committee’s regulations. Mice were group housed (two to five siblings) in a temperature-controlled environment (22–24°C), had *ad libitum* access to food and water, and were reared in normal lighting conditions (12/12 h light/dark cycle). Mice of either sex were used, except in the case of behavioral experiments, which included only male mice.

*Gad67-GFP* (MGI ID: 3590301; Tamamaki et al., 2003), *I12b-Cre* (MGI ID: 3833422; Potter et al., 2009), and *Ai14* (MGI ID: 3809524; Madisen et al., 2010) mice were maintained on a mixed background outcrossed to CD-1. Wild-type CD-1 mice were used for electrophysiology experiments. *Dlx5/6*^+/−^ mice (MGI ID: 5583955; Robledo et al., 2002) were backcrossed to C57Bl/6 mice for at least six generations for behavioral experiments.

### Epigenomic experiments

#### Histone modification chromatin immunoprecipitation and sequencing (ChIP-seq)

Immature CINs and non-CINs were isolated from P2 *Gad67-GFP* heterozygotes for native ChIP for histone posttranslational modifications. The pups were anesthetized on ice and decapitated. Neocortical tissue was dissected in ice-cold Earle’s balanced saline solution (EBSS) and cut into small pieces. The tissue was dissociated using the Papain Dissociation System (Worthington) according to the manufacturer’s instructions. After addition of the inhibitor solution, the tissue was gently aspirated 10–15 times with a P1000 pipette and filtered through a 40-µm filter to achieve a single-cell suspension. The cells were spun down and resuspended in EBSS for fluorescence-activated cell sorting (FACS) on a FACSAria II machine (BD Biosciences). Sorted GFP-positive CINs and GFP-negative non-CINs were collected separately into DMEM with 20% fetal bovine serum (FBS).

The cells were washed once then resuspended in buffer 1 (0.3 m sucrose, 60 mm KCl, 15 mm NaCl, 5 mm MgCl_2_, 0.1 mm EGTA, 15 mm Tris-HCl pH7.5, 1 mm DTT, 0.1 mm PMSF, 10 mm sodium butyrate, and 1× EDTA-free protease inhibitors). The cells were lysed by adding an equal volume of buffer 2 (buffer 1 + 0.4% v/v NP-40) and incubating on ice for 7 min. Nuclei were spun down and resuspended in 0.32 m sucrose, 50 mm Tris-HCl, pH7.5, 4 mm MgCl_2_, 1 mm CaCl_2_, 0.1 mm PMSF, and 10 mm sodium butyrate. Micrococcal nuclease was added for 6 min at 37°C to cleave chromatin into mononucleosome and dinucleosome fragments, and the reaction was stopped by addition of 20 mm EDTA. Nuclei were spun down, and the supernatant containing the soluble S1 chromatin fraction was saved. Nuclei were resuspended in dialysis buffer (1 mm Tris-HCl, pH 7.5, 0.2 mm EDTA, 0.2 mm PMSF, 10 mm Na butyrate, and 1× EDTA-free protease inhibitors) and rotated overnight at 4°C. After spinning down the supernatant containing the S2 chromatin fraction was combined with the S1 fraction for ChIP.

Histone modification ChIP was performed with antibodies against H3K27ac (Millipore catalog #05-1334, RRID:AB_1977244) or H3K27me3 (Millipore catalog #07-449, RRID:AB_310624). Chromatin from ∼80,000 nuclei was used for each condition. Antibody/chromatin complexes were purified using Dynabeads (Invitrogen) and washed extensively in Wash buffer 1 (50 mm Tris-HCl, pH 7.5, 10 mm EDTA, 125 mm NaCl, 5 mm sodium butyrate, and 0.1% v/v Tween 20), Wash buffer 2 (50 mm Tris-HCl, pH 7.5, 10 mm EDTA, 125 mm NaCl, 5 mm sodium butyrate, and 0.1% v/v NP-40), and TE. Complexes were eluted in 1% SDS, 10 mm sodium bicarbonate buffer at 65°C for 10 min. Eluted chromatin was treated with RNase (10 mg/200 ml reaction, 15 min at 37°C) and Proteinase K (10 mg/200 ml reaction, 60 min at 55°C) and cleaned using a ChIP DNA Clean & Concentrator kit (Zymo Research catalog #D5205). DNA libraries were prepared using the Ovation Ultralow DR Multiplex System (NuGEN Technologies) according to the manufacturer’s instructions. The libraries were size selected (180- to 300-bp range) using the BluePippin cassette system (Sage Science) and analyzed on a Bioanalyzer HS DNA chip (Agilent). The libraries were sequenced as single-ended 50-nucleotide reads on a HiSeq 4000 (Illumina).

#### ChIP-qPCR

H3K27ac enrichment was validated by ChIP-qPCR. H3K27ac ChIP was performed on sorted CIN and non-CIN cells as described above, and the purified chromatin was used for qPCR. We performed triplicate reactions in 10 µl volumes using the PerfeCTa SYBR Green 2× FastMix (Quanta Biosciences) on a CFX384 Real Time PCR system (Bio-Rad). The primers used are shown in Table 1. Triplicate Ct values were averaged, and fold-enrichment over matched input controls was calculated using the 2^-ΔΔCt^ method (Livak and Schmittgen, 2001): Ct values were first normalized to a *Dlx2* upstream non-conserved control region (*Dlx2-non*), i.e., ΔCt(sample) = Ct(target) – Ct(*Dlx2-non)*; ChIP DNA samples were then normalized to the corresponding input DNA, i.e., ΔΔCt = ΔCt(ChIP) – ΔCt(input). Finally, the fold enrichment for each CIN sample was divided by fold enrichment for the matched non-CIN sample and log_2_ transformed, resulting in positive fold change values for CIN-enriched loci and negative values for non-CIN enriched loci.

**Table 1:** Primer sequences used for ChIP-qPCR

Target	Forward primer	Reverse primer
*Dlx2* non-conserved control region*	5′-CAGGACTAAGCAGGCCTTTG-3′	5′-TGACCCCAATGACTCTCCAC-3′
*Gad2_En*	5′-TGCGTAGGTGCACTTAGCTTT-3′	5′-CTCAGTCCTGTACTGCTCCTTA-3′
*hs1533*	5′-GGTGGCATGTGGTGGTTCTAT-3′	5′-CGGGGCAAAGACAGACGAT-3′
*hs1175*	5′-CTCGTTGGCACAGAAACGGA-3′	5′-GGGCCCTTGTGGAAATGTTG-3′
*hs1060*	5′-TGCTTGCTTTGGCTACCGTAA-3′	5′-GCAGTGTAATTCCTTGTGACAGTT-3′
*hs1172*	5′-AATGCTCGCCACACTTCAATG-3′	5′-GCAATTCGGGCAAAGTGGATT-3′
I12b*	5′-CGGGCCCATCAAACACAAC-3′	5′-TGGGCGAAAAAATTGCTCAT-3′
*Arl4d_RE*	5′-TTTGCATCTAAGGGGCCGAC-3′	5′-CGCCCTAGAGAGAACTCACC-3′
*Dlgap1_RE*	5′-ACCGATGATGACCGTAAAGGG-3′	5′-AGTGATGACCGCCAGAAGGA-3′

*Lindtner et al. (2019).

**Table 2:** Primer sequences used for viral vector cloning

Fragment	Forward primer	Reverse primer
*Arl4d_RE*	5’-ggttcctgcggccgcacgcgtATGTTAAACAATCTTTATACCAATCCC-3’	5′-CTGCAGGAACCGGTACTAGTTGCAGATTTTCAGCCTCCCA-3′
*Dlgap1_RE*	5’-ggttcctgcggccgcacgcgtCTCTAGATTATCAGTTTTATCCCC-3’	5′-CTGCAGGAACCGGTACTAGTATCAGTACTTATGGGAAAAGATAAAC-3′
*Bg* min promoter (for *Arl4d)*	5′-AATCTGCAACTAGTACCGGTTCCTGCAGCCCGGGCTGGGC-3′	5′-GGAGTCGACTCTAGAGGATCCGCCGCGCTCTGCTTCTGGA-3′
*Bg* min promoter (for *Dlgap1)*	5′-GTACTGATACTAGTACCGGTTCCTGCAGCCCGGGCTGGGC-3′	Same as above

##### Transcription factor (TF) ChIP

Embryonic day (E)13.5 basal ganglia TF ChIP-seq data were downloaded from the NCBI GEO database: DLX2 (accession: GSE124936; Lindtner et al., 2019), LHX6 (accession: GSE85704), and NKX2-1 (accession: GSE85704; Sandberg et al., 2016).

#### Sequence alignment

Histone modification ChIP and TF ChIP sequencing reads were aligned to the mouse genome (mm10) using BWA (v0.7.15) *mem* (Li, 2013). Histone modification ChIP replicates were aligned separately for further processing (see Histone modification ChIP peak calling and postprocessing). We also generated replicate-merged histone modification ChIP-seq alignments for visualization in the main figures. The two DLX2 replicates had already been merged (Lindtner et al., 2019); we merged the three LHX6 and NKX2-1 replicates during alignment for consistency with the DLX2 TF ChIP data. We used samtools (v1.10) to convert the resulting SAM files to BAM files as well as handle the MAPQ filtering and sorting (Li et al., 2009). We used a quality filter of 30.

To visualize our reads on the UCSC Genome Browser we generated bigwig files using deepTools (v3.4.3) *bamCoverage* (Ramírez et al., 2016). We used a bin size of 10 and a fragment extension length of 200. We also normalized to RPKM and ignored duplicate reads.

#### Histone modification ChIP peak calling and postprocessing

For our histone samples, treating replicates as separate samples, we identified significant peaks against matched input controls using MACS2 (v2.1.1) *callpeak* (Zhang et al., 2008). We disabled model-based peak calling and local significance testing and used a fixed fragment extension length of 200 bp. We used broad peak calling on both H3K27ac and H3K27me3 ChIP-seq samples with a broad-cutoff of 0.01 and a *q* value cutoff of 0.01.

We then tested the consistency of our replicates using the irreproducible discovery rate (IDR) R package (v1.2; Li et al., 2011), using the settings suggested in their package description (μ = 2.6, σ = 1.3, ρ = 0.8, *p* = 0.7; https://cran.r-project.org/web/packages/idr/idr.pdf). We used log10 fold-change scores from MACS2 as our input to calculate the IDR scores.

After confirming good distributions of signal-to-noise in our replicates, we then identified differential peaks between the CIN and non-CIN peak sets for each replicate using the MACS2 differential peak calling (bdgdiff) method with a minimum peak length of 150 bp (Zhang et al., 2008). This identified three sets of peaks for each replicate: CIN-enriched, non-CIN-enriched and common peaks.

Within each single bed file, peaks separated by 500 bp or less were merged to reduce variation in peak definitions because of coverage differences across the complex regions of enrichment often seen in histone modification ChIP-seq (unlike TF peaks, which tend to be discrete peaks) using bedtools merge (Quinlan and Hall, 2010). Finally, we defined high-confidence (HC) peak sets for each condition by finding the intersection between peak sets for the two replicates using bedtools intersect (Quinlan and Hall, 2010).

#### TF ChIP-seq postprocessing

For our TF ChIP-seq samples, we also used MACS2 (v2.1.1) *callpeak* (Zhang et al., 2008). We disabled model-based peak calling and local significance testing. We used a fixed fragment extension length of 200 bp and a *q* value cutoff of 0.01.

We merged our DLX2, LHX6, and NKX2-1 TF ChIP-seq peaks to form a set of the union of the three TFs and a set of the intersect of all three TFs. We then used the bedtools intersect method to locate the overlapping regions between our set of differentially called H3K27ac ChIP-seq peaks and our TF ChIP-seq sets.

#### ATAC-seq and conservation data

We obtained previously published chromatin accessibility data from ATAC-seq on purified CINs and PNs from GEO (accession: GSE63137; Mo et al., 2015). We used the provided bigwigs and called peaks without further processing. Bigwigs from replicate 1 are shown in the figures Cconserved elements predicted by phastCons (60-way vertebrate conservation) were obtained through the UCSC browser (https://genome.ucsc.edu).

#### Genomic annotation and motif analysis

We used HOMER (Heinz et al., 2010) to call motifs in our peak sets, using a size window of 200. We used the findMotifsGenome.pl and annotatePeaks.pl methods to acquire sets of enriched TF binding motifs for all our experiments and genomic annotations for our peaks. We also used the -genomeOntology setting to acquire genome ontology tables. TF binding motif fold-enrichment was calculated as the % of targets/% of background.

Gene-region associations and Gene Ontology (GO) analysis were performed on the HC peak sets with Genomic Regions Enrichment of Annotations Tool (GREAT) v4.0 (http://great.stanford.edu/public/html/index.php) using the default basal+extension association rule (McLean et al., 2010). GO term significance was based on default settings of binomial and hypergeometric false discovery rate (FDR) < 0.05 and region fold enrichment > 2.

### Data availability

The histone modification ChIP-seq data generated in this study have been deposited in the NCBI GEO database and are accessible through GEO Series accession number GSE158428.

### AAV methods

#### AAV production

The plasmid *(p)AAV-I12b-Bg-ChR2-EYFP* was reported previously (Cho et al., 2015). *pAAV-I12b-Bg-ChR2-EYFP* was modified, with the *Arl4d* and *Dlgap1* enhancers replacing *I12b*, using Gibson assembly cloning (Gibson et al., 2009). Briefly, *Arl4d_RE* and *Dlgap1_RE* were amplified from mouse genomic DNA with the addition of overlap sequences using the primers in Table 2. The *Beta-globin* (*Bg*) minimal promoter region was amplified from *pAAV-I12b-Bg-ChR2-EYFP*. AgeI and SpeI cut sites were also introduced 3′ to the enhancers and 5′ to the *Bg* minimal promoter. Finally, *pAAV-I12b-Bg-ChR2-EYFP* was cut with MluI and BamHI, and the enhancer, *Bg* promoter and backbone were assembled for each plasmid using the In-Fusion HD Cloning kit (Takara Bio USA) according to the manufacturer’s instructions. Recombinant AAVs were packaged with serotype AAV5 by Virovek, Inc. AAV5-hSyn-ChR2-EYFP was obtained from the UNC Vector Core.

#### Virus injections

Adult mice were anesthetized using isoflurane (2.5% induction, 1.2–1.5% maintenance, in 95% oxygen) and placed in a stereotaxic frame (David Kopf Instruments). Body temperature was maintained using a heating pad. An incision was made to expose the skull for stereotaxic alignment using bregma and lambda as vertical references. For the *Dlx5/6*^+/−^ mice for behavioral experiments, the scalp and periosteum were removed from the dorsal surface of the skull and scored with a scalpel to improve implant adhesion.

The mice were injected in the mPFC near the border between the prelimbic and infralimbic cortices (1.7 anterior-posterior, ±0.3 mediolateral, and −2.6 dorsoventral mm relative to bregma) with AAV-Arl4d-ChR2-EYFP, AAV-Dlgap1-ChR2-EYFP, AAV-I12b-ChR2-EYFP or AAV-hSyn-ChR2-EYFP (600–1000 nl per hemisphere). Viruses were infused at 100–150 nL/min through a 35-gauge, beveled injection needle (World Precision Instruments) using a microsyringe pump (World Precision Instruments, UMP3 UltraMicroPump). After infusion, the needle was kept at the injection site for 5–10 min and then slowly withdrawn. After surgery, mice were allowed to recover until ambulatory on a heated pad, then returned to their homecage. Approximately eight weeks after injection, wild-type mice were used for electrophysiological characterization, *I12b-Cre;Ai14* mice were used for immunohistochemistry and cell counts, and *Dlx5/6*^+/−^ mice were used for behavioral experiments.

### Electrophysiology methods

#### Acute cortical slice preparation

Mice were anesthetized with an intraperitoneal injection of euthasol and transcardially perfused with an ice-cold cutting solution containing the following: 210 mm sucrose, 2.5 mm KCl, 1.25 mm NaH_2_PO_4_, 25 mm NaHCO_3_, 0.5 mm CaCl_2_, 7 mm MgCl_2_, and 7 mm dextrose (bubbled with 95% O_2_-5% CO_2_, pH ∼7.4). Approximately, three coronal slices (250 µm thick) of the brain containing the mPFC were obtained using a vibrating blade microtome (VT1200S, Leica Microsystems Inc.). Slices were allowed to recover at 34°C for 30 min followed by 30-min recovery at room temperature in a holding solution containing the following: 125 mm NaCl, 2.5 mm KCl, 1.25 mm NaH_2_PO_4_, 25 mm NaHCO_3_, 2 mm CaCl_2_, 2 mm MgCl_2_, 12.5 mm dextrose, 1.3 mm ascorbic acid, and 3 mm sodium pyruvate.

#### Whole-cell patch clamp recordings

Somatic whole-cell current clamp and voltage clamp recordings were obtained from submerged slices perfused in heated (32–34°C) artificial CSF (aCSF) containing the following: 125 mm NaCl, 3 mm KCl, 1.25 mm NaH_2_ PO_4_, 25 mm NaHCO_3_, 2 mm CaCl_2_, 1 mm MgCl_2_, and 12.5 mm dextrose (bubbled with 95% O_2_/5% CO_2_, pH ∼7.4). Neurons were visualized using DIC optics fitted with a 40× water-immersion objective (BX51WI, Olympus microscope). PNs and EYFP-expressing CINs located in layer 5 were targeted for patching. Patch electrodes (2–4 MΩ) were pulled from borosilicate capillary glass of external diameter 1 mm (Sutter Instruments) using a Flaming/Brown micropipette puller (model P-2000, Sutter Instruments). For current clamp recordings, electrodes were filled with an internal solution containing the following the following: 120 mm K-gluconate, 20 mm KCl, 10 mm HEPES, 4 mm NaCl, 7 mm K_2_-phosphocreatine, 0.3 mm Na-GTP, and 4 mm Mg-ATP (pH ∼7.3 adjusted with KOH). Biocytin (Vector Laboratories) was included (0.1–0.2%) for subsequent histologic processing. For voltage clamp recordings, the internal solution contained the following the following: 130 mm Cs-methanesulfonate, 10 mm CsCl, 10 mm HEPES, 4 mm NaCl, 7 mm phosphocreatine, 0.3 mm Na-GTP, 4 mm Mg-ATP, and 2 mm QX314-Br (pH ∼7.3 adjusted with CsOH).

Electrophysiology data were recorded using Multiclamp 700B amplifier (Molecular Devices). Voltages have not been corrected for measured liquid junction potential (∼8 mV). Upon successful transition to the whole-cell configuration, the neuron was given at least 5 min to stabilize before data were collected. During current clamp recordings, series resistance and pipette capacitance were appropriately compensated. Series resistance was usually 10–20 MΩ, and experiments were terminated if series resistances exceeded 25 MΩ.

#### Electrophysiology protocols and data analysis

All data analyses were performed using custom routines written in IGOR Pro (Wavemetrics).

Resting membrane potential (RMP) was measured as the membrane voltage measured in current clamp mode immediately after reaching the whole-cell configuration. Input resistance (Rin) was calculated as the slope of the linear fit of the voltage-current plot generated from a family of hyperpolarizing and depolarizing current injections (−50 to +20 pA, steps of 10 pA). Firing output was calculated as the number of action potentials (APs) fired in response to 800-ms-long depolarizing current injections (25–500 pA). Firing frequency was calculating as the number of APs fired per second. Rheobase was measured as the minimum current injection that elicited spiking. Firing traces in response to 50-pA current above the rheobase were used for analysis of single AP properties: AP threshold, maximum *dV*/*dt* (rate of rise of AP), AP amplitude, AP half-width and fast afterhyperpolarization (fAHP) amplitude. Threshold was defined as the voltage at which the value of third derivative of voltage (with respect to time) is maximum. AP amplitude was measured from threshold to peak, with the half-width measured at half this distance. fAHP was measured from the threshold to the negative voltage peak after the AP. Index of spike-frequency accommodation (SFA) was calculated as the ratio of the last inter-spike interval to the first inter-spike interval. Recorded CINs were classified as fast-spiking or regular-spiking based on electrophysiological properties. Specifically, CINs were classified as fast spiking if they satisfied at least three of the following four criteria: AP half-width < 0.5 ms, max firing frequency > 50 Hz, fAHP amplitude > 15 mV, or SFA < 2.

To measure optogenetically evoked spiking in EYFP^+^ CINs, and optogenetically evoked IPSCs in layer 5 PNs, we stimulated ChR2 using 5 ms long single light pulses (light power: 4 mW/mm^2^) generated by a lambda DG-4 high-speed optical switch with a 300 W Xenon lamp (Sutter Instruments) and an excitation filter centered around 470 nm, delivered to the slice through a 40× objective (Olympus).

### Histology

#### *Post hoc* immunohistochemistry (IHC) and biocytin labeling

Patched slices were fixed overnight in 4% PFA then transferred to PBS. All incubations were conducted on a rotating platform at room temperature unless stated otherwise. The sections were rinsed twice in PBS, then blocked and permeabilized for 3 h in PBS with 10% FBS, 0.5% Triton X-100, and 0.05% sodium azide. Sections were immunostained overnight with primary antibodies: rabbit anti-PV (1:1000, Swant catalog #PV-27, RRID:AB_2631173) and rat anti-GFP (1:1000, Nacalai Tesque catalog #04404-84, RRID:AB_10013361) in PBS with 0.1% Triton X-100, 10% FBS and 0.025% sodium azide. Sections were washed 2 × 30 min in PBS with 0.25% Triton X-100, and 2 × 30 min in PBS. Goat anti-rabbit Alexa Fluor 488 secondary antibody (1:750, Invitrogen, catalog #A-11034) and Streptavidin-647 (1:500, Invitrogen, catalog #S-32357) were added with Hoescht 33 342 nuclear counterstain (1:2000, Invitrogen, catalog #H3570) for 4–6 h at room temperature, then overnight at 4°C. After washing 2 × 30 min in PBS with 0.25% Triton X-100, and 2 × 30 min in PBS, sections were mounted on Superfrost Plus slides and coverslipped with Fluorescence Mounting Medium (DAKO catalog #S3023).

#### EYFP and CIN marker colocalization

AAV-injected *I12b-Cre; Ai14* mice were anesthetized with an intraperitonial injection of ketamine/xylazine and transcardially perfused with ice-cold PBS followed by fixative solution (4% PFA in 0.24 m sodium phosphate buffer, pH 7.2). Brains were dissected out and postfixed in fixative solution overnight at 4°C, followed by cryopreservation for 24 h in 15% sucrose in PBS and 24 h in 30% sucrose in PBS. Coronal sections of 40-µm thickness were obtained using a freezing microtome (SM2000 R, Leica Microsystems Inc.) and stored in sectioning solution (40% PBS, 30% glycerol, 30% ethylene glycol) at −20°C until use.

Sections were immunostained using a standard floating section IHC protocol with gentle agitation on a rotary shaking platform. Briefly, sections were rinsed in PBS, blocked for 1 h at room temperature in PBST (PBS + 0.25% Triton X-100) with 10% FBS, and incubated with primary antibody diluted in PBST + 10% FBS overnight at 4°C. Primary antibodies used were: rabbit anti-PV (1:1000, Swant catalog #PV-27, RRID:AB_2631173), rat anti-SST (1:400, Millipore catalog #MAB354, RRID:AB_2255365), rabbit anti-VIP (1:250, ImmunoStar catalog #20077, RRID:AB_572270), rat anti-GFP (1:1000, Nacalai Tesque catalog #04404-84, RRID:AB_10013361), and rabbit anti-GFP (1:5000, Abcam catalog #ab6556, RRID:AB_305564). The following day, sections were washed 2 × 15 min in PBST and 2 × 15 min in PBS, incubated for 2 h at room temperature with Alexa Fluor-conjugated secondary antibodies (1:750, Invitrogen). Finally, sections were washed 2 × 15 min in PBST and 2 × 15 min in PBS, mounted on Superfrost Plus slides and coverslipped with Fluorescence Mounting Medium (DAKO catalog #S3023).

##### Imaging

Low-magnification epifluorescent images were taken using a Coolsnap camera (Photometrics) mounted on a Nikon Eclipse 80i microscope using NIS Elements acquisition software (Nikon). Confocal images were taken with 20× air and 40× oil objectives on an Andor Borealis CSU-W1 spinning disk confocal mounted on a Nikon Ti Microscope and captured with an Andor Zyla sCMOS camera and Micro-Manager software (Open Imaging). The raw images were preprocessed with ImageJ software (v2.0.0) to adjust brightness/contrast and convert to eight-bit RGB. Confocal *z*-stacks were stitched laterally to create composite stacks using the Grid/Collection stitching ImageJ plugin with linear blending (Preibisch et al., 2009).

#### Cell counts

Cell counting was performed in Adobe Photoshop CC on a single stitched 20× confocal image plane. EYFP^+^ and marker^+^ cells were counted in the mPFC at the rostrocaudal levels shown in Extended Data Figure 4-1. For each virus, at least three rostrocaudal sections were counted for each of at least two animals. EYFP or marker-positive cells were counted using only the corresponding single-color channel before determining colocalization of markers among these cells. Counts are presented as % (EYFP^+^ marker^+^ cells/total EYFP^+^ cells). Counts normalized to CIN subtype abundance are also presented, i.e., (EYFP^+^ marker^+^ cells/total EYFP^+^ cells) ÷ (tdTomato^+^ marker^+^ cells/total tdTomato^+^ cells). The normalized counts for AAV-hSyn-ChR2-EYFP injected animals include EYFP^+^ CINs only (co-expressing tdTomato), rather than the total EYFP^+^ population.

**Figure 1.**
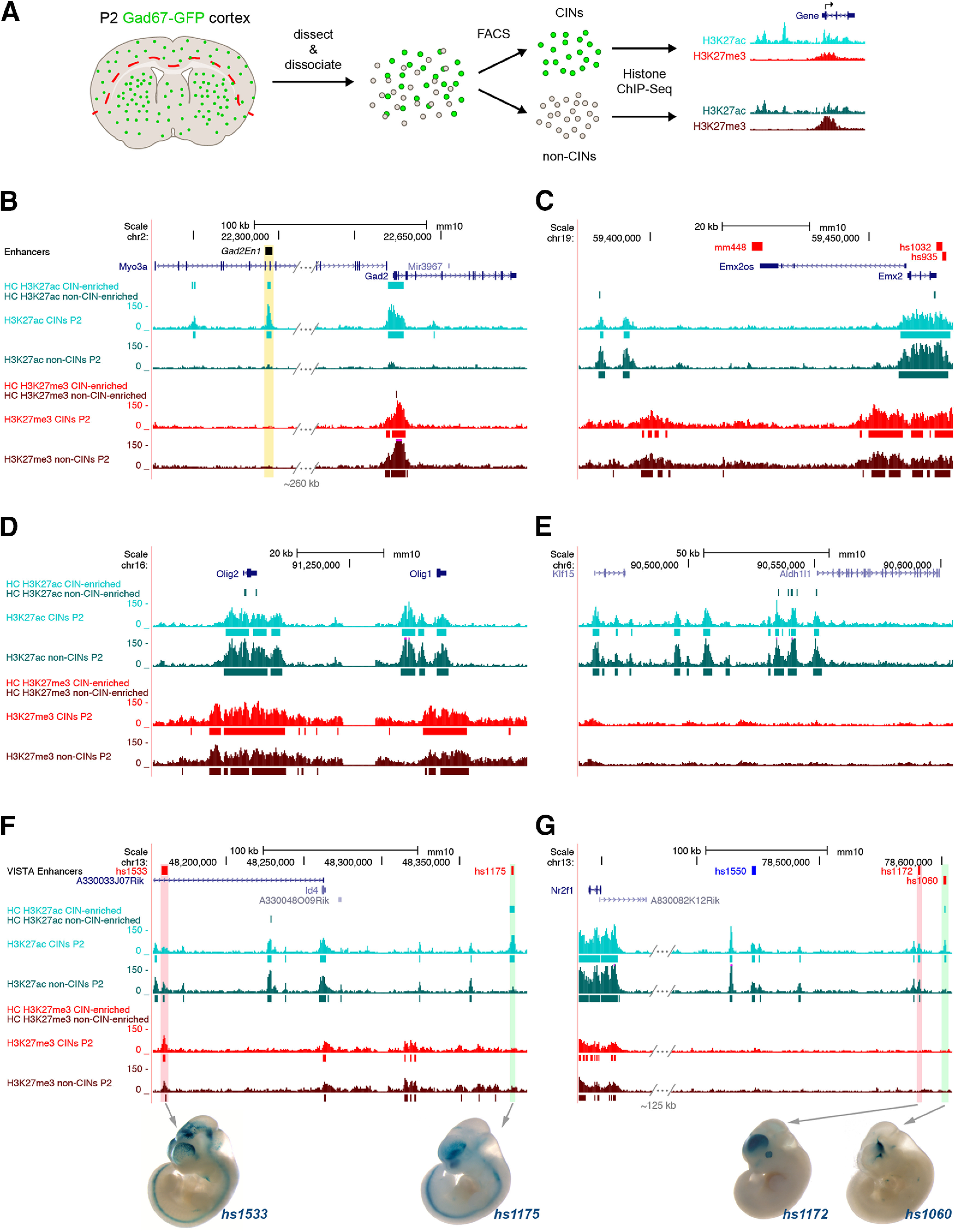
ChIP-seq reveals differential enrichment of histone modifications in P2 CINs and non-CINs. ***A***, Schematic showing the histone modification ChIP-seq workflow using CINs and non-CINs dissected and FACS-purified from P2 *Gad67-GFP* neocortex. ***B–E***, Enrichment of H3K27ac (active mark, cyan and teal) and H3K27me3 (repressive mark, red and dark red) around genes expressed in CINs (*Gad2*), PNs (*Emx2*) oligodendrocyte-lineage cells (*Olig1, Olig2*), and astrocytes (*Aldh1l1*). *Gad2En1* is a validated enhancer with activity in adult CINs (yellow highlight; Pla et al., 2017). ***F***, ***G***, H3K27ac enrichment is similar in CINs and non-CINs across the gene body for *Id4* and *Nr2f1* but differentially enriched at enhancers with validated activity in the basal ganglia (green highlights) or cortex (red highlights). Whole-mount transient transgenics from the VISTA browser show the pattern of enhancer activity at E11.5 (https://enhancer.lbl.gov; Visel et al., 2013). ChIP-qPCR validation of H3K27ac enrichment at highlighted enhancers is shown in Extended Data Figure 1-1. HC called peaks are shown as solid bars under the corresponding bigwig tracks.

10.1523/ENEURO.0211-20.2020.f1-1 Extended Data Figure 1-1 Validation of histone modification ChIP-seq. ***A***, IDR analysis showing replicate reproducibility for histone modification ChIP-seq. ***B***, Table detailing the number of called peaks from histone modification ChIP-seq experiments by biological replicate. HC peaks were those called for both replicates. ***C***, Validation of H3K27ac enrichment at REs and pREs by ChIP-qPCR, expressed as log_2_ fold change of CIN enrichment over non-CIN enrichment. Fold changes were not significantly different to zero by one-sample *t* test (*p *>* *0.05). Download Figure 1-1, EPS file.

### Rule shift methods

#### Surgery

Male *Dlx5/6*^+/−^ adult mice (15 weeks old at time of experiment) received bilateral injections of AAV-Arl4d-ChR2-EYFP into the mPFC as described above. After injection of virus, a 200/240 mm (core/outer) diameter, NA = 0.22, dual fiber-optic cannula (Doric Lenses, DFC_200/240–0.22_2.3 mm_GS0.7_FLT) was slowly inserted into mPFC until the tip of the fiber reached a dorsoventral depth of −2.25 mm relative to bregma. Implants were affixed onto the skull using Metabond Quick Adhesive Cement (Parkell). We waited at least eight weeks after injection before behavioral experiments to allow for virus expression.

#### Rule shift task

For the cognitive flexibility task, mice were singly-housed and habituated to a reverse light/dark cycle and food intake was restricted until the mice reached 80–85% of the *ad libitum* feeding weight. At the start of each trial, the mouse was placed in its home cage to explore two bowls, each containing one odor and one digging medium, until it dug in one bowl, signifying a choice. As soon as a mouse began to dig in one bowl, the other bowl was removed, so there was no opportunity for “bowl switching.” The bait was a piece of a peanut butter chip (∼5–10 mg in weight) and the cues, either olfactory (odor) or somatosensory and visual (texture of the digging medium which hides the bait), were altered and counterbalanced. All cues were presented in small animal food bowls (All Living Things Nibble bowls, PetSmart) that were identical in color and size. Digging media were mixed with the odor (0.01% by volume) and peanut butter chip powder (0.1% by volume). All odors were ground dried spices (McCormick garlic and coriander), and unscented digging media (Mosser Lee White Sand Soil Cover, Natural Integrity Clumping Clay cat litter).

After mice reached their target weight, they underwent 1 d of habituation. On this day, mice were given 10 consecutive trials with the baited food bowl to ascertain that they could reliably dig and that only one bowl contained food reward. All mice were able to dig for the reward. Then, on days 1 and 2, mice performed the task (this was the testing done for experiments). After the task was done for the day, the bowls were filled with different odor-medium combinations and food was evenly distributed among these bowls and given to the mouse so that the mouse would disregard any associations made earlier in the day.

Mice were tested through a series of trials. The determination of which odor and medium to pair and which side (left or right) contained the baited bowl was randomized (subject to the requirement that the same combination of pairing and side did not repeat on more than three consecutive trials) using http://random.org. On each trial, while the particular odor-medium combination present in each of the two bowls may have changed, the particular stimulus (e.g., a particular odor or medium) that signaled the presence of food reward remained constant over each portion of the task (initial association and rule shift). If the initial association paired a specific odor with food reward, then the digging medium would be considered the irrelevant dimension. The mouse is considered to have learned the initial association between stimulus and reward if it makes eight correct choices during 10 consecutive trials. Each portion of the task ended when the mouse met this criterion. Following the initial association, the rule shift portion of the task began, and the particular stimulus associated with reward underwent an extradimensional shift. For example, if an odor had been associated with reward during the initial association, then a digging medium was associated with reward during the rule shift portion of the task. The mouse is considered to have learned this extradimensional rule shift if it makes eight correct choices during 10 consecutive trials. When a mouse makes a correct choice on a trial, it is allowed to consume the food reward before the next trial. Following correct trials, the mouse is transferred from the home cage to a holding cage for ∼10 s while the new bowls were set up (intertrial interval). After making an error on a trial, a mouse was transferred to the holding cage for ∼2 min (intertrial interval). All animals performed the initial association in a similar number of trials (average: 10–15 trials).

#### *In vivo* optogenetic stimulation

A 473 nm blue laser (OEM Laser Systems) was coupled to the dual fiber-optic cannula (Doric Lenses) through a 200 mm diameter dual fiber-optic patchcord with guiding socket (Doric Lenses) and 1 × 2 intensity division fiber-optic rotary joint (Doric Lenses), and adjusted such that the final light power was ∼0.5 mW total, summed across both fibers and averaged over light pulses and the intervening periods. A function generator (Agilent 33500B Series waveform Generator) connected to the laser generated a 40 Hz train of 5 ms pulses.

Light stimulation began once mice reached the 80% criterion during the initial association portion of the task. Mice then performed three additional initial association trials before the rule shift portion of the task began.

### Statistical analysis

Statistical analyses were performed using Prism 7 (GraphPad) and detailed in the corresponding figure legends. Student’s *t* tests were used to make single-variable comparisons. For multivariate comparisons, we used one-way ANOVA with Tukey’s corrected *p* values for multiple comparisons. Chi-square or Fisher’s exact tests were used to test for significance among contingency-type data; *p < 0.05, **p < 0.01, ***p < 0.001, ****p < 0.0001. Comparisons with no asterisk had *p *>* *0.05 and were considered not significant.

## Results

### Identification of candidate CIN gene REs

To identify putative REs (pREs) that could be used to drive expression in immature CINs, and potentially in mature CINs, we used genome-wide epigenetic profiling to identify regions of the genome that have signatures of an active transcriptional state (Fig. 1*A*). We used FACS to separate GFP^+^ immature CINs and GFP^-^ non-CIN cells (enriched for glutamatergic PNs and glia) from acutely dissected and dissociated cortical tissue from postnatal day (P)2 *Gad67-GFP* heterozygote pups. Chromatin was extracted from the purified cell populations and processed for native histone modification ChIP with antibodies raised against H3K27ac, a mark of active REs, and H3K27me3, a mark of poised and repressive REs, followed by next-generation DNA sequencing (Creyghton et al., 2010; Rada-Iglesias et al., 2011). Genomic regions with enrichment (“peaks”) of H3K27ac and H3K27me3 were called by comparing the ChIP samples with an input control. IDR analysis confirmed high concordance between all pairs of replicates (Extended Data Fig. 1-1*A*; Li et al., 2011).

We thus defined high-confidence (HC) peaks as those present in both biological replicates. A total of 35,768 HC H3K27ac peaks were called for the CIN samples and 34,953 for the non-CINs. These peaks represent the pREs that could be driving gene expression in these cells, including proximal REs (promoters) and distal REs (enhancers). We then performed differential peak calling to identify regions with significantly more H3K27ac in CINs versus non-CINs, and vice versa. By this method, we identified 2937 HC CIN-specific pREs and 718 HC non-CIN pREs; 25,556 HC peaks were common to both populations. The HC peak sets were used for all downstream analyses. Peak numbers by replicate can be found in Extended Data Figure 1-1*B*.

A total of 14,915 HC H3K27me3 peaks were called for CIN samples and 17,550 were called for non-CINs. These peaks represent putatively poised and/or repressed REs, which would be expected to be associated with lower expression of their target genes. By differential peak calling, we identified 168 HC regions with more H3K27me3 in CINs than non-CINs and 285 HC regions with higher H3K27me3 in non-CINs. Peak numbers by replicate can be found in Extended Data Figure 1-1*B*.

**Figure 2.**
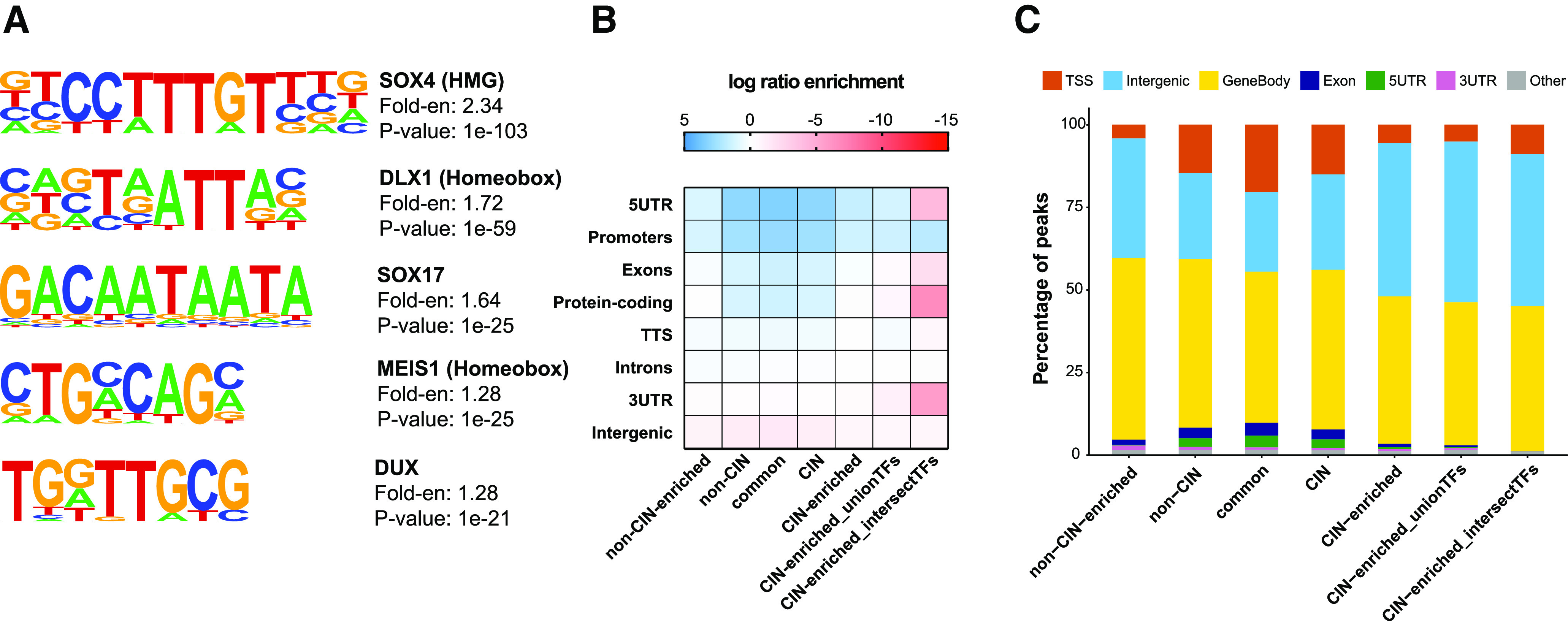
Motif analysis and genomic annotation of regions enriched for histone H3K27ac. ***A***, *De novo* motif analysis of CIN-enriched H3K27ac peaks by HOMER, showing the top five enriched motifs, their best-match known motifs, fold-enrichment (Fold-en) over background, and significance of enrichment. *De novo* motif analyses of other peak sets are shown in Extended Data Figure 2–1*A–C*. ***B***, Heatmap showing the enrichment of genomic annotations (curated list) generated by HOMER for the different sets of H3K27ac peaks compared with background. The full list of annotations and enrichment values are shown in Extended Data Figure 2-1*D*. CIN-enriched_unionTFs peaks are CIN-enriched peaks that overlap with at least one DLX2, LHX6, or NKX2-1 ChIP binding site. CIN-enriched_intersectTFs peaks are CIN-enriched peaks that overlap with DLX2, LHX6, and NKX2-1 binding sites. ***C***, Proportion of peaks in each set associated with genomic annotations generated by HOMER. GO analyses of genes associated with the CIN-enriched, non-CIN-enriched, and common peaks are shown in Extended Data Figures 2-1*E*,*F*, 2-2. TTS: transcription termination site, TSS: transcription start site, UTR: untranslated region.

We validated our histone ChIP-seq data first by confirming patterns of histone modification enrichment at known marker gene loci. As expected, CIN-relevant genes (e.g., *Dlx1*, *Dlx2*, *Gad1*, and *Gad2*) had significant enrichment of H3K27ac (greater activation) and depletion of H3K27me3 (less repression) in CINs compared with non-CINs (Figs. 1*B*, 2*A***;** Extended Data Fig. 2-1*B*). Conversely, markers for PNs (*Emx2*), oligodendrocytes (*Olig1* and *Olig2*) and astrocytes (*Aldh1l1*) showed greater enrichment of H3K27ac over the gene body and proximal sequences in the non-CIN samples (Fig. 1*C–E*). Statistically significant differential enrichment of H3K27ac was also found at validated enhancers (distal REs) active in CINs or their progenitors in the basal ganglia. For example, H3K27ac was enriched specifically in CINs at *Gad2En1*, *hs1175* near *Id4*, and *hs1060* near *Nr2f1* (Fig. 1*B*,*F*,*G*; Visel et al., 2013; Silberberg et al., 2016; Pla et al., 2017). Conversely, H3K27ac was enriched in one of the two non-CIN samples at *hs1533* near *Id4*, and *hs1172* near *Nr2f1* (Fig. 1*F*,*G*), which have cortical activity at E11.5 (Visel et al., 2013; Pattabiraman et al., 2014).

10.1523/ENEURO.0211-20.2020.f2-1 Extended Data Figure 2-1 Motif analysis and genomic annotation of regions enriched for histone H3K27ac. ***A****–****C***, *De novo* motif analysis by HOMER, showing the top five enriched motifs, their best-match known motifs, fold-enrichment over background, and significance of enrichment. ***D***, Genomic ontology generated by HOMER, showing log ratio enrichment over background. Significantly overrepresented (or underrepresented) annotations (*p *<* *0.01) are shown in bold. ***E***, ***F***, GO analysis of gene-associated H3K27ac-enriched regions by GREAT for CIN-specific and non-CIN peaks. The top ten significantly enriched biological process terms are shown for each peak set ranked by binomial *p* value. Relevant terms are highlighted in cyan. The full list of enriched GO biological process terms can be found in Extended Data Figure 2-2. Download Figure 2-1, EPS file.

10.1523/ENEURO.0211-20.2020.f2-2 Extended Data Figure 2-2 Full lists of significantly enriched GO terms from GREAT analysis for the HC common, CIN-specific, and non-CIN H3K27ac peaks. Download Figure 2-2, XLSX file.

We then confirmed differential enrichment of H3K27ac at these REs by ChIP-qPCR on sorted CIN and non-CIN samples from P2 *Gad67-GFP* cortices. *Gad2En1*, *hs1175*, and *hs1060* showed a trend for enrichment in the CIN samples, while *hs1533* and *hs1172* were more enriched in non-CINs as expected (Extended Data Fig. 1-1*C*). However, these enrichments were not significant by one-sample *t* test (*p *>* *0.05).

We hypothesized that REs specifically active in CINs would be bound by TFs with known roles in CIN development. Therefore, we performed *de novo* motif discovery with HOMER to identify TF binding motifs enriched in the CIN-enriched H3K27ac peak set and compared these to motifs enriched in the non-CIN-enriched and common peaks (Fig. 2*A*; Extended Data Fig. 2-1*A*,*B*).

The top CIN-enriched motifs included SOX motifs, a DLX motif and a MEIS motif and were highly significant (*p *≤* *1e-21; Fig. 2*A*). DLX TFs are central to GABAergic cell development (Hu et al., 2017; Lindtner et al., 2019). MEIS1 is transcriptionally downstream of DLX1/2 and ARX (another important CIN TF) and is expressed in CINs migrating to the cortex (Long et al., 2009; Friocourt and Parnavelas, 2011). The enrichment of these binding motifs suggests that the CIN-enriched peaks represent functional REs that may regulate CIN gene expression. The non-CIN-enriched *de novo* motifs had low *p* values (≤1e-11) and were flagged as possible false positives by HOMER (Extended Data Fig. 2-1*A*). They were also found in <5% of the target sequences, compared with 17–43% of target sequences for the top five CIN-enriched motifs. This suggests a lack of any strong consensus TF binding motifs among the non-CIN-enriched peak set, which may be a function of the cell type heterogeneity of the non-CIN sample.

To determine whether CIN-enriched and non-CIN-enriched peaks are associated with particular functional regions of the genome, we also performed genomic annotation with HOMER. All the peak sets (CIN, non-CIN, CIN-enriched, non-CIN-enriched and common peaks) showed enrichment over background at promoters, 5′ untranslated regions (5′UTRs) and introns, as expected for H3K27ac ChIP (Fig. 2*B*; Extended Data Fig. 2-1*D*).

Compared with common (not differentially enriched) peaks, both CIN-enriched and non-CIN-enriched peaks were less often associated with promoter regions/transcription start sites (TSSs), exons and 5′UTRs, and more often associated with intergenic regions (Fig. 2*C*). This supports the notion that chromatin activation state is more dynamically regulated at enhancers than promoters, and, therefore, enhancers may show more cell type-specific activity than promoters.

We then used the GREAT to assign H3K27ac peaks to nearby genes they may regulate (McLean et al., 2010). GO analysis of gene-associated H3K27ac-enriched regions with GREAT showed significant enrichment of relevant biological process GO terms for each population. The top ten GO terms for CIN-specific pREs included terms related to forebrain neurogenesis and specifically GABAergic CIN differentiation, while terms further down the list were also associated with neuronal migration and axonogenesis (Extended Data Figs. 2-1*E*, 2-2). HC non-CIN pREs were strongly associated with glial development (Extended Data Fig. 2-1*F*). The significantly enriched GO terms for the common peak set were overwhelmed by terms related to generic cellular processes such as transcription and translation (Extended Data Fig. 2-2). However, many genes related to synaptogenesis and pan-neuronal development and function were associated with multiple common H3K27ac peaks (e.g., *Tubb2a/b*, *Nlgn1/2/3*, *Ctnnb1*, *Rbfox2*, and *Camk2d*).

To refine our list of candidate pREs, we leveraged previously published TF ChIP-seq data from embryonic basal ganglia (Sandberg et al., 2016; Lindtner et al., 2019). DLX2, LHX6, and NKX2-1 are TFs important for CIN development, whose expression within the telencephalon is primarily limited to the GABAergic lineages generated in the basal ganglia anlage (Kessaris et al., 2014; Hu et al., 2017). We hypothesized that DLX2, LHX6, and NKX2-1 binding at pREs increases the probability that the RE will be active in maturing GABAergic lineage neurons including CINs.

A total of 40% of CIN-specific pREs (1163 out of 2937) overlapped with at least one called DLX2, LHX6, or NKX2-1 peak (CIN-enriched_unionTF peak set). The most significantly enriched *de novo* motif found at CIN-enriched_unionTF pREs (TAATTACVVS) was a close match for known DLX1/2/5/6 motifs, and also very similar to published LHX6 consensus motifs (Extended Data Fig. 2-1*C*; Sandberg et al., 2016).

Sequence conservation across species is another predictor of functional REs (Nord et al., 2015). We found that 72% (2115 out of 2937) of our HC CIN-specific pREs overlapped with predicted vertebrate conserved elements identified by phastCons (obtained through the UCSC browser). Furthermore, chromatin accessibility is a prerequisite for RE activation and gene expression. We therefore integrated published chromatin accessibility data from ATAC-seq on sorted adult cortical cells to further inform our selection of REs to test (Mo et al., 2015).

**Figure 3.**
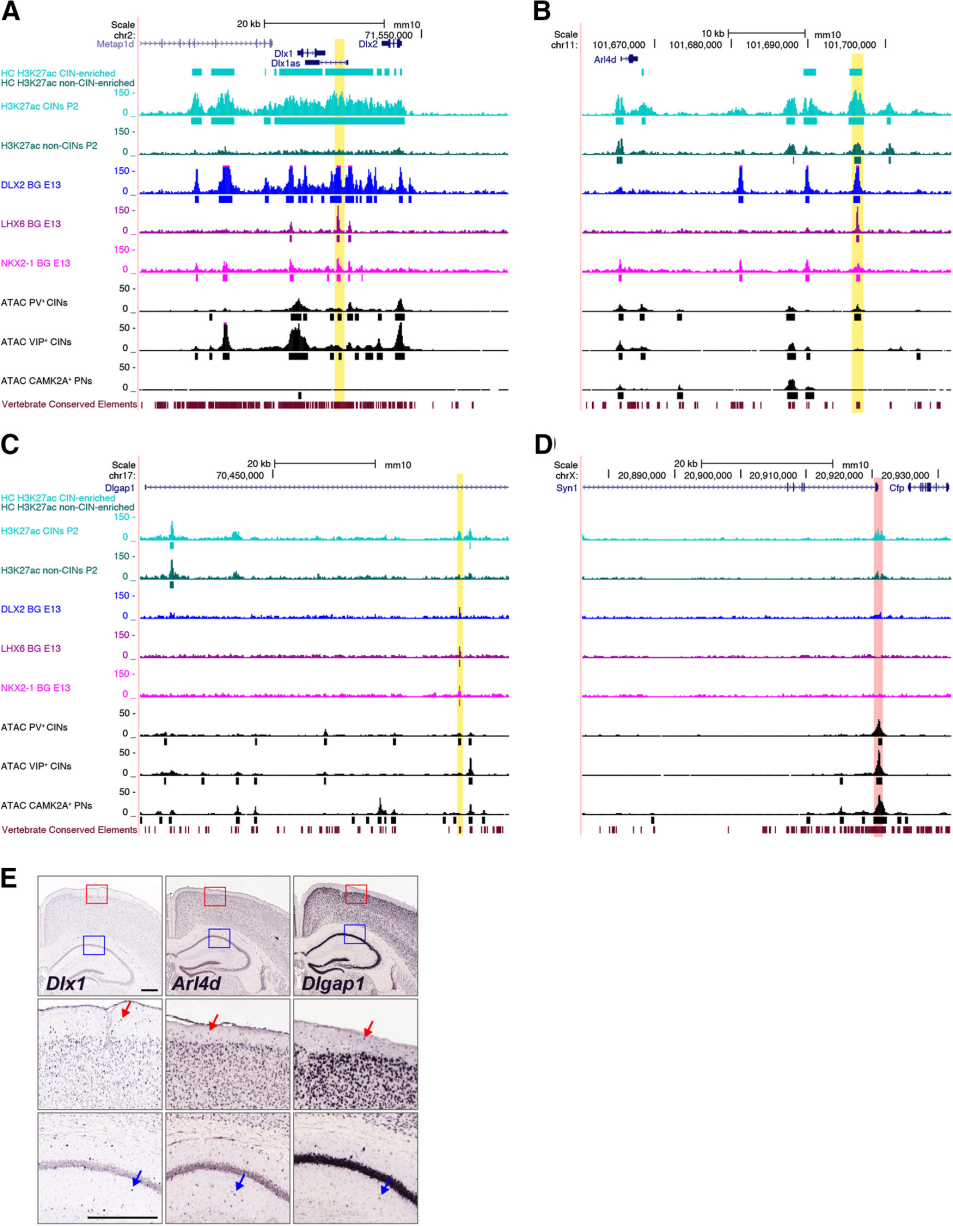
Integrated epigenetic signatures of candidate CIN pREs. ***A***, The *I12b* RE region (yellow highlight) is highly enriched for H3K27ac in CINs compared with the non-CINs. It also has Dlx2, LHX6, and NKX2-1 TF binding peaks in E13.5 basal ganglia (BG) and shows enhanced accessibility by ATAC-seq in P30 PV^+^ and VIP^+^ CINs, but not in CAMK2A^+^ PNs. ***B***, The *Arl4d* pRE (yellow highlight) is highly enriched for H3K27ac in CINs compared with non-CINs, has DLX2, LHX6, and NKX2-1 TF binding peaks and shows enhanced accessibility by ATAC-seq in P30 PV^+^ CINs but not VIP^+^ CINs or CAMK2A^+^ PNs. It also overlaps a vertebrate conserved element. ***C***, The *Dlgap1* intronic pRE (yellow highlight) shows greater enrichment of H3K27ac in CINs than in non-CINs and modest binding peaks for DLX2, LHX6, and NKX2-1. The *Dlgap1* pRE overlaps an ATAC peak in P30 PV^+^ CINs, but not in VIP^+^ CINs or PNs, and a vertebrate conserved element. ***D***, The *Syn1* promoter region (red highlight) has comparable H3K27ac enrichment in CINs and non-CINs and is highly accessible in P30 PV^+^ CINs, VIP^+^ CINs and CAMK2A^+^ PNs. The highly conserved homologous human region drives AAV expression in many PNs and some CINs (see Fig. 5). HC called peaks are shown as solid bars under the corresponding bigwig track. Additional pREs are shown in Extended Data Figure 3-1. Coverage and called peaks separated by replicate for the *Arl4d* and *Dlgap1* loci are shown in Extended Data Figure 3-2. ***E***, *In situ* hybridization in adult cortex shows CIN-specific staining of *Dlx1*. *Arl4d* and *Dlgap1* mRNA appear to be expressed in CINs [especially obvious in neocortical layer 1 (red arrows) and hippocampal stratum radiatum (blue arrows)] and many PNs. Images are taken from the Allen Brain Atlas (https://www.brain-map.org; Lein et al., 2007). Scale bars: 500 µm.

As a proof of principle, we looked at the *I12b* enhancer, which is located between *Dlx1* and *Dlx2*. *I12b* has well-characterized activity in the ganglionic eminences and their derivates, including CINs, in transgenic mice and when placed in viral vectors (Potter et al., 2009; Arguello et al., 2013; Lee et al., 2014b; Cho et al., 2015; Vogt et al., 2015). We found that the entire *Dlx1, Dlx2* and *I12b* genomic region was highly enriched for H3K27ac in the purified P2 CINs, but not in the non-CIN population (Fig. 3*A*). *I12b* was also bound by DLX2, LHX6, and NKX2-1 in E13.5 basal ganglia cells based on TF ChIP (Fig. 3*A*). Additionally, ATAC-seq demonstrated that the region was accessible in adult PV^+^ and VIP^+^ CINs, but not in purified excitatory CAMK2A^+^ PNs (Fig. 3*A*; Mo et al., 2015).

10.1523/ENEURO.0211-20.2020.f3-1 Extended Data Figure 3-1 Epigenetic signatures of additional candidate CIN pREs. ***A***, The *Arx* locus contains several validated enhancers. Enhancers with activity in the basal ganglia (green highlights) have HC H3K27ac CIN-specific peaks, DLX2, LHX6, and NKX2-1 TF binding peaks, and enhanced accessibility by ATAC-seq in P30 PV^+^ and/or VIP^+^ CINs but not in CAMK2A^+^ PNs. Enhancers active in the cortex (red highlights) or outside the telencephalon (blue highlights) lack this particular signature: *hs123* is also accessibly by ATAC in CAMK2A^+^ PNs; *hs122* has H3K27ac enrichment in both CINs and non-CINs. Sections showing transient transgenic enhancer activity at E11.5 are taken from the VISTA Enhancer Browser (https://enhancer.lbl.gov; Visel et al., 2013). ***B****–****F***, Epigenetic signatures of CIN-specific pREs (yellow highlights) near the CIN genes *Gad1*, *Slc32a1* (*Vgat*), *Htr3a*, *Maf*, and *Sst*. Download Figure 3-1, TIF file.

10.1523/ENEURO.0211-20.2020.f3-2 Extended Data Figure 3-2 H3K27ac enrichment at pREs separated by replicate. Browser tracks showing merged coverage and HC called peaks (white background), and coverage and called peaks by replicate (grey background) for CINs and non-CINs. Differential called peaks (CIN-enriched or non-CIN-enriched) are shown at the top. ***A***, The *Arl4d* pRE (yellow highlight) shows strong H3K27ac enrichment in CINs in both replicates and is called as CIN-enriched in both replicates. ***B***, The *Dlgap1* pRE (yellow highlight) shows modest H3K27ac enrichment, with a called CIN peak and differential CIN-enriched peak in replicate 1. Download Figure 3-2, EPS file.

Based on the positive results with the *I12b* locus, we screened other loci for similar epigenetic signatures. The *Arx* locus contains several REs with validated enhancer activity in the developing telencephalon at E11.5: *hs119* and *hs121* are active in the basal ganglia, while *hs122* and *hs123* are active in cortical progenitors (VISTA REs, https://enhancer.lbl.gov; Visel et al., 2013)*. hs121* (also known as UAS3) also remains active in immature and adult CINs (Colasante et al., 2008). All four enhancers had enrichment of DLX2 TF binding in E13.5 basal ganglia, but only the REs active in the basal ganglia and immature CINs had both HC CIN-specific H3K27ac peaks at P2 and chromatin accessibility in adult PV^+^ or VIP^+^ CINs but not in CAMK2A^+^ PNs (Extended Data Fig. 3-1*A*). This highlights the utility of integrating multiple epigenetic marks to identify strong CIN-specific pRE candidates.

Among the many possibilities we focused on pREs located near two genes: *Arl4d* and *Dlgap1*. Both *Arl4d* and *Dlgap1* mRNA are expressed in the embryonic basal ganglia (the ganglionic eminences), and their expression is downregulated on loss of *Nkx2-1* expression in the medial ganglionic eminence (Sandberg et al., 2016). *Arl4d* expression is also reduced in the basal ganglia of *Dlx1/2* knock-outs (Lindtner et al., 2019). In the adult, *Arl4d* is expressed in many cells throughout the cortex, including CINs most visible in neocortical layer 1 and the hippocampus (Fig. 3*E*). DLGAP1 is expressed at excitatory postsynaptic densities in neurons throughout the forebrain (Fig. 3*E*; Rasmussen et al., 2017). Although both of these genes show widespread expression in forebrain neurons, enhancers are often active in a restricted subset of their target gene’s expression domain (Visel et al., 2013).

The *Arl4d* pRE is located ∼30 kb downstream of the *Arl4d* TSS. H3K27ac enrichment was significantly higher in P2 CINs compared with non-CINs (Fig. 3*B*). The locus also contains TF binding peaks for DLX2, LHX6, and NKX2-1 and had ATAC-seq peaks in adult PV CINs but not in VIP CINs or PNs (Fig. 3*B*). The *Dlgap1* pRE is located within its first intron. This locus showed modest enrichment of H3K27ac in P2 CINs (called in one replicate, see Extended Data Fig. 3-2*B*), as well as binding peaks for DLX2, LHX6, and NKX2-1 in embryonic basal ganglia (Fig. 3*C*). Both the *Arl4d* and *Dlgap1* pREs also overlapped with vertebrate conserved elements predicted by phastCons, suggesting their activity could translate across species.

H3K27ac enrichment was further validated by ChIP-qPCR. *I12b*, the *Arl4d* pRE, and the *Dlgap1* pRE all showed relative enrichment in CINs versus non-CINs (Extended Data Fig. 1-1*C*). The magnitude of enrichment was similar for the three REs, although it was not significant by one-sample *t* test (*p *>* *0.05).

Many further candidate CIN pREs were identified based on these integrated epigenetic data. Several examples found near the CIN-expressed genes *Gad1*, *Slc32a1* (*Vgat*), *Htr3a*, *Maf*, and *Sst* are shown in Extended Data Figure 3-1*B–F*. The epigenetic signature at the mouse *Syn1* promoter, homologous to the human region used as a control RE active in PNs (see CIN RE AAVs preferentially label PV-positive neurons in the mPFC), did not contain any HC H3K27ac peaks or called TF binding peaks but was highly accessible in PV and VIP CINs as well as in PNs by ATAC-seq (Fig. 3*D*).

### Physiologic properties of neurons targeted by the RE AAVs

CINs can be classified into diverse subclasses based on molecular markers, morphology, circuit function and electrophysiological properties (Kepecs and Fishell, 2014; Tremblay et al., 2016). To test whether our candidate pREs (*Arl4d_RE* and *Dlgap1_RE*) can be used to generate viral tools for the observation and manipulation of physiologically distinct subpopulations of CINs, we made AAV constructs using these pREs to drive expression of a channelrhodopsin-enhanced YFP fusion protein (ChR2-EYFP). The resulting AAVs (AAV-Arl4d-ChR2-EYFP and AAV-Dlgap1-ChR2-EYFP) and a previously characterized RE AAV (AAV-I12b-ChR2-EYFP; Cho et al., 2015) were injected into the deep layers of the mPFC.

We obtained current clamp recordings from transduced layer 5 neurons, identified by EYFP expression (Extended Data Fig. 4-1*A*). Reliable short latency (<10 ms) firing in response to flashes of blue light (470 nm) was used to validate AAV-mediated ChR2 expression in the recorded EYFP^+^ CINs (Extended Data Fig. 4-1*E*). Notably, the RE AAVs specifically targeted CINs and not PNs in layer 5 of the mPFC, as optogenetic stimulation of RE AAV-labeled neurons elicited robust IPSCs in EYFP negative layer 5 PNs, which were completely blocked by bath application of 10 μm gabazine (GABA_A_ receptor antagonist; Extended Data Fig. 4-1*F*).

**Figure 4.**
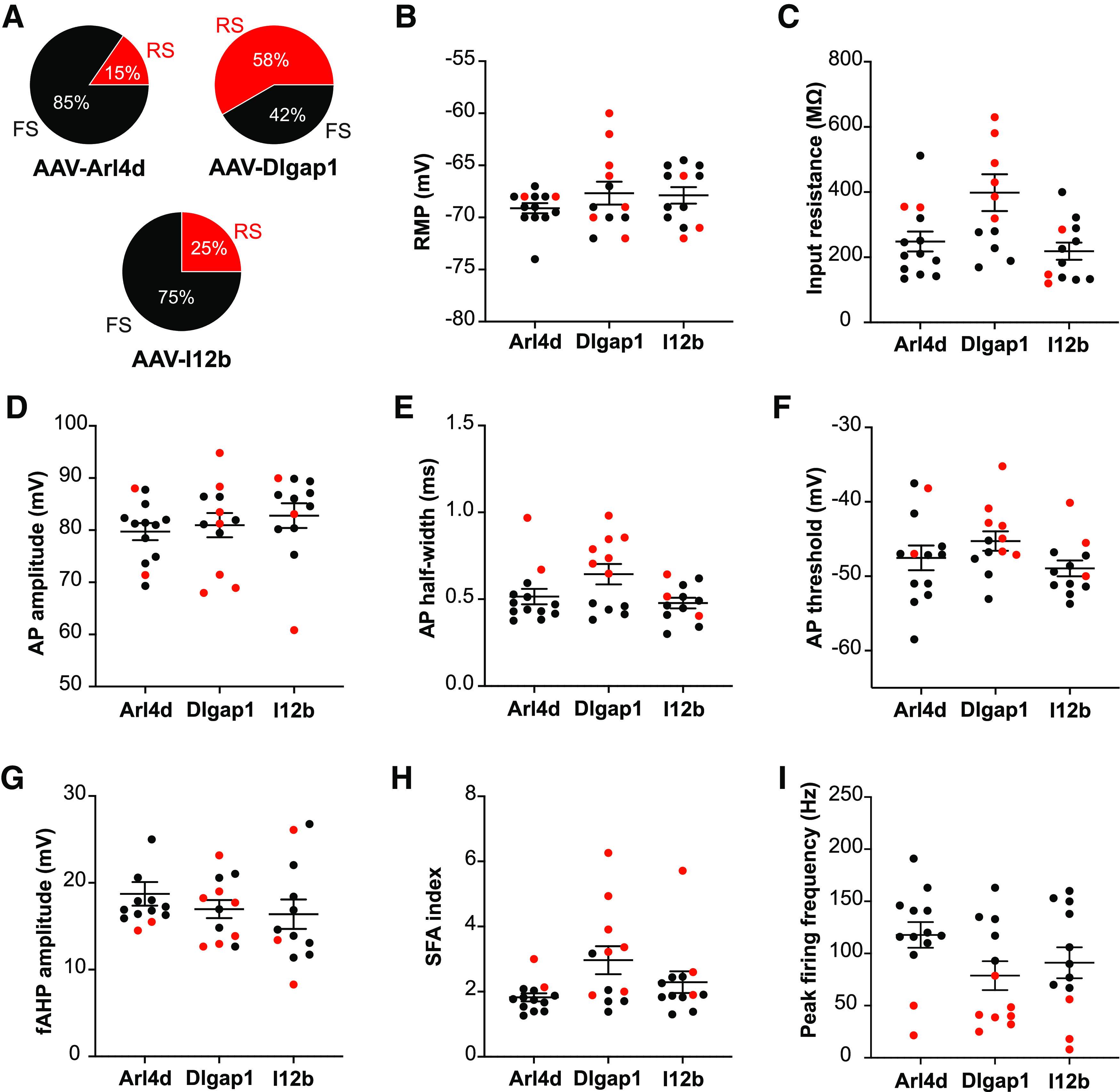
Electrophysiological properties of CINs targeted by AAV-Arl4d, AAV-Dlgap1, and AAV-I12b. ***A***, Pie charts showing the percentages of fast-spiking (FS) and regular-spiking (RS) CINs targeted by the different pREs. AAV-Arl4d-ChR2-EYFP: 11/13 FS, 2/13 RS; AAV-Dlgap1-ChR2-EYFP: 5/12 FS, 7/12 RS; AAV-I12b-ChR2-EYFP: 9/12 FS, 3/12 RS. ***B–I***, Subthreshold, AP, and firing properties of the layer 5 CINs targeted by the enhancer AAVs. Black dots denote FS CINs, and red dots denote RS CINs. Data are shown as mean ± SEM. Additional methodology and *post hoc* immunolabeling of recorded cells is shown in Extended Data Figure 4-1.

To determine whether different RE AAVs differentially target CINs with distinct physiological properties, recorded cells were classified as either fast spiking or regular spiking based on their firing output and AP properties (Extended Data Fig. 4-1*B–D*). This analysis showed that a large proportion of CINs targeted by AAV-Arl4d-ChR2-EYFP had fast-spiking properties (11/13 neurons; Fig. 4*A*). Specifically, the fast-spiking CINs targeted by AAV-Arl4d-ChR2-EYFP had shorter AP half-width, large fAHP amplitude, low SFA and high peak firing frequency in comparison to the smaller proportion of EYFP^+^ regular-spiking CINs (Extended Data Fig. 4-1*E–I*). Similar to the transduction specificity of AAV-Arl4d-ChR2-EYFP, a large proportion of AAV-I12b-ChR2-EYFP labeled CINs also had fast-spiking physiological properties (9/12 neurons; Fig. 4). In contrast, AAV-Dlgap1-ChR2-EYFP labeled a larger proportion of regular-spiking CINs (7/12 neurons) than fast-spiking CINs (5/12 neurons; Fig. 4). Overall, the proportions of regular versus fast-spiking CINs transduced by AAV-Arl4d-ChR2-EYFP versus AAV-Dlgap1-ChR2-EYFP were significantly different (*p *=* *0.0414, Fisher’s exact test).

10.1523/ENEURO.0211-20.2020.f4-1 Extended Data Figure 4-1 Electrophysiological characterization of layer 5 CINs targeted by RE-AAVs. ***A***, DIC and fluorescent images showing that EYFP^+^ layer 5 CINs in mPFC were targeted during patch clamp recordings. Scale bar: 25 µm. ***B***, Example voltage traces in response to subthreshold current injections (–50 to +50 pA, top) and to suprathreshold current injections (250 and 375 pA) in an EYFP^+^ fast-spiking (FS) CIN. ***C***, Same as ***B*** for an EYFP^+^ regular-spiking (RS) CIN. ***D***, Example voltage traces showing single APs recorded from EYFP^+^ FS and RS CINs. ***E***, left, Recording configuration showing that current clamp recordings were obtained from layer 5 EYFP^+^ CINs. Right, APs were reliably elicited in EYFP^+^ CINs in response to blue light (470 nm) flashes during patch clamp recordings. ***F***, left, Recording configuration showing that voltage clamp recordings (+10 mV) were obtained from EYFP-negative layer 5 PNs. IPSCs (black trace) were recorded in PNs in response to blue light flashes. Currents were completely blocked by application of gabazine (10 µm). ***G***, Representative images of *post hoc* labeling for biocytin, PV, and EYFP in recorded cells for each enhancer AAV injection. Arrows indicate PV^+^ recorded cells and open arrowhead indicates a PV^–^ recorded cell. Scale bar: 25 µm. ***H***, Pie charts showing the percentages of biocytin-labeled FS and RS CINs expressing PV by *post hoc* labeling. Some cells were not recovered after patching. Number of cells of each type is indicated. Download Figure 4-1, tif file.

Since fast-spiking physiological properties are associated with CINs that express PV (Kawaguchi et al., 1987; Rudy et al., 2011), we immunostained for PV expression in a subset of recorded EYFP^+^ neurons (Extended Data Fig. 4-1*G*,*H*). In line with the increased targeting of fast-spiking CINs by AAV-Arl4d-ChR2-EYFP and AAV-I12b-ChR2-EYFP, a large proportion of EYFP^+^ CINs transduced with these enhancer AAVs expressed PV (Arl4d: 5/5 labeled neurons; I12b: 8/12 labeled neurons). Surprisingly, many AAV-Dlgap1-ChR2-EYFP-labeled neurons also expressed PV (7/8 neurons; Fig. 4*A*; Extended Data Fig. 4-1*G*,*H*).

### CIN RE AAVs preferentially label PV-positive neurons in the mPFC

**Figure 5.**
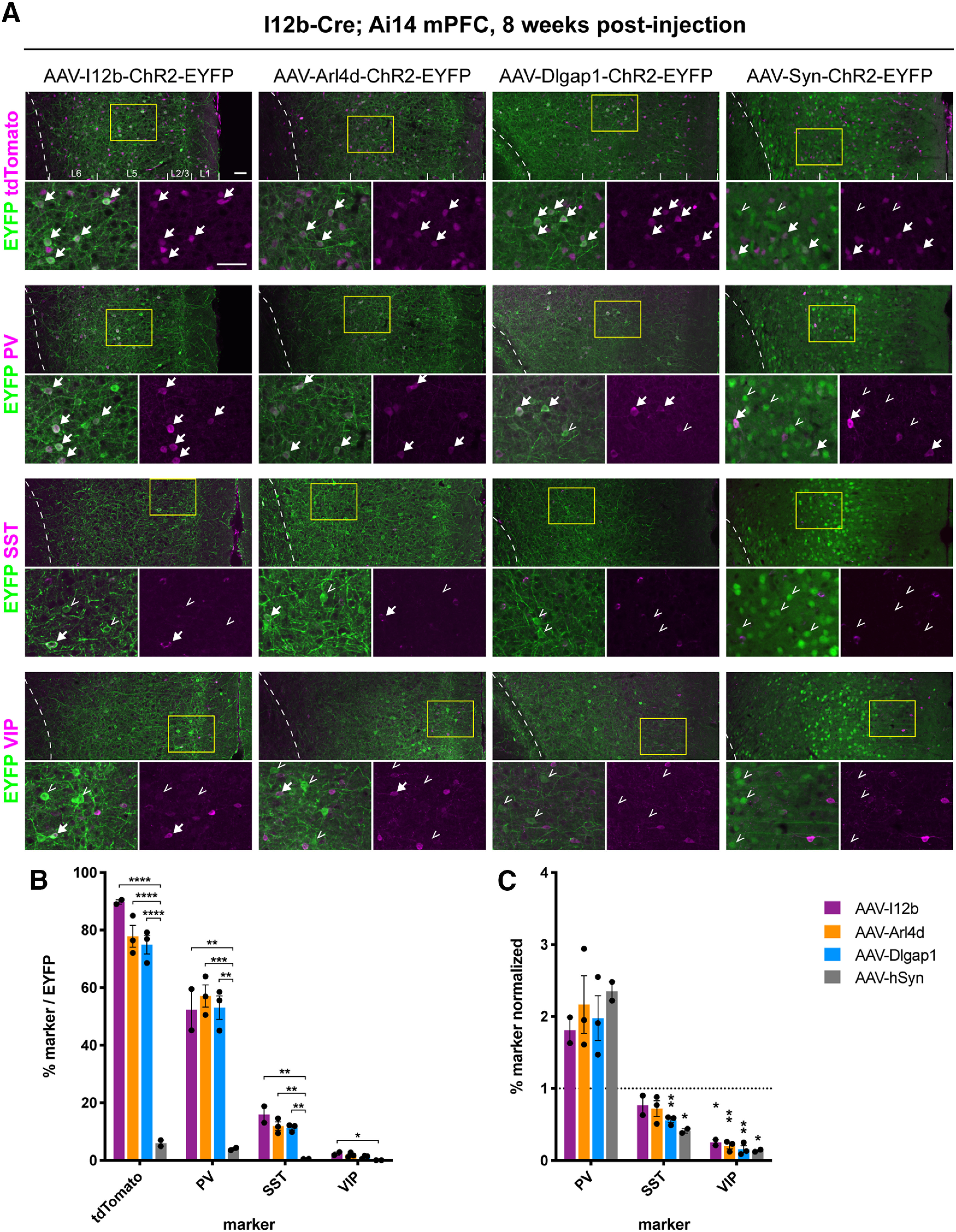
IHC characterization of CINs targeted by RE-AAVs injected into adult *I12b-Cre*; *Ai14* mPFC. ***A***, Single confocal planes showing colocalization of EYFP from the RE-AAVs with endogenous tdTomato and CIN subtype markers PV, SST, and VIP within the mPFC. tdTomato expression from the *I12b-Cre; Ai14* reporter line marks most CINs. Dotted white lines indicate border between cortical layer 6 and corpus callosum, and approximate layer markings are shown in the low-magnification tdTomato panels (top). Yellow boxes indicate areas shown at high magnification. Arrows indicate EYFP^+^, marker^+^ cells; open arrowheads indicate EYFP^+^ only cells. Scale bars: 50 µm. (tdTomato and PV images are from the same section for each animal.) ***B***, Quantification of the % of EYFP^+^ cells targeted by the RE AAVs that express tdTomato and the CIN subtype markers PV, SST, and VIP. One-way ANOVA (tdTomato: I12b vs hSyn, Arl4d vs hSyn, Dlgap1 vs hSyn; ****p <* *0.0001); (PV: I12b vs hSyn, **p =* *0.0018; Arl4d vs hSyn, ***p =* *0.0007; Dlgap1 vs hSyn, **p =* *0.0010); (SST: I12b vs hSyn, **p =* *0.0025; Arl4d vs hSyn, **p =* *0.0074; Dlgap1 vs hSyn, **p =* *0.0097); (VIP: I12b vs hSyn, *p =* *0.0488). ***C***, % of EYFP^+^ cells expressing CIN subtype markers normalized to the abundance of each subtype. Values used for normalization are summarized in Extended Data Figure 4-1*C*. A one-sample *t* test was used to determine significant difference of normalized values from 1 (SST: AAV-Dlgap1, **p =* *0.0063; AAV-hSyn *p =* *0.0323; VIP: AAV-I12b, *p =* *0.0339; AAV-Arl4d, **p =* *0.0028; AAV-Dlgap1, **p =* *0.0032; AAV-hSyn, *p =* *0.0110). Data are shown as mean ± SEM, as well as individual values for each animal. Non-significant *p* values not shown. Quantification of EYFP and marker colocalization broken down by rostro-caudal level is shown in Extended Data Figure 5-1*D–F*.

To further characterize the molecular identity of the RE AAV-labeled neurons, we injected the AAV-RE-ChR2-EYFP viruses into the mPFC of adult *I12b-Cre;Ai14* mice, in which the majority of CINs are labeled by tdTomato reporter expression (Extended Data Fig. 5-1*B*; Potter et al., 2009). As an additional control virus, we used AAV-hSyn-ChR2-EYFP, which labels both PNs and CINs. After seven to eight weeks, we performed immunohistochemistry (IHC) for EYFP and CIN subtype markers SST, PV, and VIP (Fig. 5*A*). Marker and EYFP colocalization was assessed in the mPFC of 2–3 bilaterally injected animals, compiled from three to four rostrocaudal sections from each animal (Extended Data Fig. 5-1*A*).

10.1523/ENEURO.0211-20.2020.f5-1 Extended Data Figure 5-1 IHC characterization of CINs targeted by RE-AAVs by rostrocaudal level of the mPFC of *I12b-Cre; Ai14* mice. ***A***, Coronal schematics of the sections ***A–D*** used for histology. Counts were taken from the areas highlighted in yellow. Cg, cingulate cortex; PrL, prelimbic cortex; IL, infralimbic cortex; VO, ventral orbital cortex; MO medial orbital cortex; DP, dorsal peduncular cortex; CPu, caudate putamen (striatum); cc, corpus callosum. Adapted from Paxinos and Franklin (2001). ***B***, Quantification of % coverage of the three main CIN subtypes by endogenous tdTomato expression in the mPFC of *I12b-Cre*; *Ai14* mice. Similar coverage of CIN populations by GFP in *I12b-Cre*; *Z/EG* somatosensory cortex was reported previously (Potter et al., 2009). ***C***, Quantification of the abundance of PV, SST, and VIP CINs as % of tdTomato^+^ cells in the mPFC of *I12b-Cre*; *Ai14* mice. ***D***, Quantification of the % of EYFP-positive cells targeted by the RE AAVs that are CINs, i.e., label for endogenous tdTomato. ***E***, Quantification of the % of EYFP-positive cells targeted by the RE AAVs that express the CIN subtype markers PV, SST, and VIP. Counts are shown separated by rostro-caudal levels ***A–D***. ***F***, % of EYFP-positive cells expressing CIN subtype markers normalized to the abundance of each subtype (% marker/EYFP divided by % marker/tdTomato), separated by rostro-caudal levels ***A–D***. Data are shown as mean ± SEM, as well as individual values for each animal. Download Figure 5-1, EPS file.

AAV-I12b-ChR2-EYFP reporter expression was restricted to GABAergic CINs as previously described (Cho et al., 2015); 90% of EYFP^+^ cells co-expressed tdTomato (Fig. 5*B*), despite CINs accounting for only ∼20% of the neurons in the cortex. In line with our electrophysiological characterization, AAV-Arl4d-ChR2-EYFP and AAV-Dlgap1-ChR2-EYFP also drove EYFP expression predominantly in CINs; EYFP cells were 78% and 75% tdTomato^+^, respectively (Fig. 5*B*). As expected, AAV-hSyn-ChR2-EYFP reporter expression was seen in many more PNs than CINs, and only 6% of EYFP^+^ cells were positive for tdTomato. The pattern of EYFP labeling produced by AAV-hSyn-ChR2-EYFP was significantly different from the CIN-restricted expression of the other three viruses (I12b vs hSyn, Arl4d vs hSyn, and Dlgap1 vs hSyn: ****p <* *0.0001; one-way ANOVA with Tukey’s correction for multiple comparisons).

We then immunostained with the CIN markers PV, SST, and VIP to determine whether our RE AAVs target specific subtypes of CINs. EYFP^+^ cells labeled by AAV-Arl4d-ChR2-EYFP and AAV-Dlgap1-ChR2-EYFP were 57% and 53% PV^+^, respectively, while AAV-I12b-ChR2-EYFP-labeled cells were 52% PV^+^ (Fig. 5*B*). SST-expressing neurons accounted for 11–16% of the EYFP^+^ cells and VIP-expressing neurons made up <3% of EYFP^+^ cells for all three CIN viruses (Fig. 5*B*). The majority of AAV-Syn-ChR2-EYFP-labeled cells were PNs and thus did not colocalize with PV, SST, or VIP. Counts separated by rostro-caudal position are shown in Extended Data Figure 5-1*D–F*.

To determine whether any of the RE AAVs preferentially label PV^+^ CINs, EYFP-CIN marker colocalization was normalized to the percent of marker^+^ CINs out of the total tdTomato^+^ CIN count in the same section (Fig. 5*C*; Extended Data Fig. 5-1*C*). AAV-I12b-ChR2-EYFP, AAV-Arl4d-ChR2-EYFP and AAV-Dlgap1-ChR2-EYFP-labeled populations were 1.8–twofold enriched for PV^+^ CINs compared with the overall CIN population, although this enrichment was not significant. SST CINs were significantly underrepresented in the EYFP^+^ population labeled by AAV-Dlgap1-ChR2-EYFP (*p *=* *0.0063, one-sample *t* test), and VIP CINs were underrepresented in the populations labeled by all three CIN viruses (I12b, *p *=* *0.0339; Arl4d, *p *=* *0.0028; Dlgap1, *p *=* *0.0032). This suggests some degree of preferential labeling of PV^+^ CINs by all three viruses.

### RE AAV-driven expression in a CIN-dependent behavioral task: rule shift

We next investigated the function of CINs targeted by the *Arl4d* pRE using a cognitive flexibility assay in which mice learn a “rule shift.” This task requires mice to choose between bowls using odor-based or texture-based cues to find hidden food rewards and then shift from an odor-based rule to a texture-based one, or vice versa. These rule shifts depend on prefrontal γ oscillations, which are believed to be generated by fast-spiking PV^+^ CINs (Cardin et al., 2009; Sohal et al., 2009; Cho and Sohal, 2014; Cho et al., 2015).

**Figure 6.**
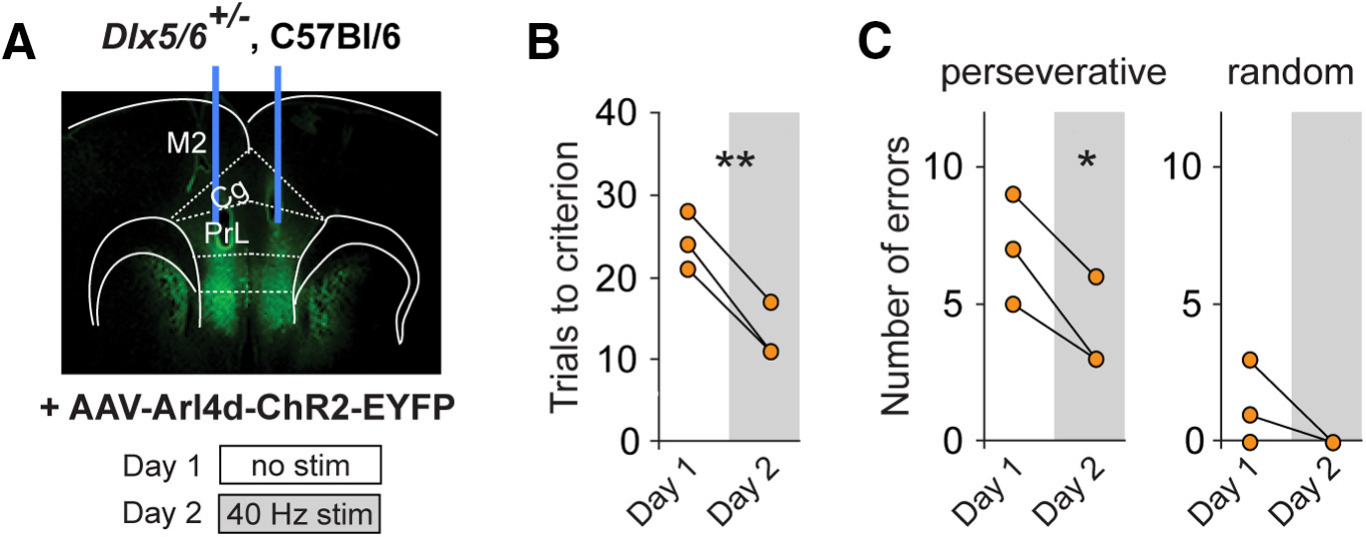
Optogenetic stimulation of AAV-Arl4d-ChR2-EYFP-transduced neurons improves cognitive flexibility in *Dlx5/6*^−/−^ mice. ***A***, *Dlx5/6*^+/−^ mice (*n* = 3) had bilateral AAV-Arl4d-ChR2-EYFP injections and fiber-optic implants into the mPFC. Low-magnification image of EYFP expression after bilateral injection of AAV-Arl4d-ChR2-EYFP. Experimental design: day 1: no stimulation; day 2: 40-Hz stimulation during the rule-shift portion of the task. ***B***, 40-Hz stimulation on day 2 rescues rule-shift behavior in mutant mice (*t*_(2)_ = 12.85, **p =* *0.006). ***C***, 40-Hz stimulation on day 2 decreased the number of perseverative errors compared with no stimulation on day 1 (*t*_(2)_ = 5.196, *p =* *0.035). There was no change in random errors (*t*_(2)_= 1.512, *p *=* *0.2697). Two-tailed, paired *t* tests were used. Individual values for each animal are shown.

In this study we took advantage of *Dlx5/6*^+/−^ mice, which have abnormal PV CIN physiology, decreased task-evoked γ oscillations, and fail to suppress perseverative responses during rule shifts (Cho et al., 2015). It was previously shown that optogenetic stimulation of mPFC CINs using AAV-I12b-ChR2-EYFP can completely normalize rule-shift performance in *Dlx5/6*^+/−^ mice. This effect occurs when stimulation is delivered at γ frequencies (40 or 60 Hz; Cho et al., 2015). Therefore, we tested whether another RE AAV that preferentially targets fast-spiking PV CINs, AAV-Arl4d-ChR2-EYFP, can also produce ChR2 expression that rescues rule-shift performance in *Dlx5/6*^+/−^ mice (Fig. 6*A*). On day 1, we assayed rule-shift performance in the absence of optogenetic stimulation to quantify baseline performance of these mutant mice. As expected, the mice were specifically impaired during the rule-shift portion of the task, making a large number of perseverative errors (Fig. 6*B*,*C*). The number of trials to criterion and perseverative errors were very similar to previously published results for adult *Dlx5/6*^+/−^ mice (Cho et al., 2015). Strikingly, delivery of optical stimulation at 40 Hz on day two dramatically improved rule-shift performance compared with day 1 in *Dlx5/6*^+/−^ mice and significantly reduced the number of perseverative errors (Fig. 6*B*,*C*). The improvements were comparable to those seen with stimulation after injection of AAV-I12b-ChR2-EYFP in the *Dlx5/6*^+/−^ mutant mice, whereas stimulation following injection of mock AAV (AAV-I12b-mCherry) did not rescue this behavioral deficit (Cho et al., 2015).

## Discussion

Elegant combinatorial transgenic approaches have made it possible to study the functions of defined cell populations *in vivo* in mice, including for subtypes of CINs (Dymecki et al., 2010; Taniguchi et al., 2011; He et al., 2016). However, these approaches cannot be easily adopted across species. The number of cell populations that can be independently accessed in the same animal is also limited with these approaches.

AAV targeting strategies have been lauded as an alternative to overcome these obstacles and to facilitate translation to human genetic therapies, but the lack of AAVs that can target specific cell types remains a significant impediment to realizing these goals within the heterogeneous cellular environment of the brain (Betley and Sternson, 2011). Here we have reported two novel REs (*Arl4d_RE* and *Dlgap1_RE*) that can be used to drive CIN expression of reporter and effector genes using the AAV delivery system. We also identified nearly three thousand CIN-specific pREs genome wide, providing a starting point for the development of further tools for CIN functional studies.

AAV vectors are restricted in their genomic carrying capacity, which limits the lengths of sequences that can be used to direct the expression of reporters and effectors. Several attempts have been made to use short promoter sequences from neuronal marker genes. This strategy has been successful for pan-neuronal or pan-excitatory neuron targeting with a variety of viral vectors, but less successful for labeling subtypes of neurons, including CINs (Kügler et al., 2003a,b; Dittgen et al., 2004; Nathanson et al., 2009a; Sohal et al., 2009). Distal REs (enhancers) can also be short enough to be used in AAV vectors. REs can drive cell-type-specific expression within the developing forebrain (Visel et al., 2013; Pattabiraman et al., 2014; Silberberg et al., 2016), and REs of the *Dlx* genes have been used in AAV to successfully target CINs in adult mice (Lee et al., 2014a,b; Cho et al., 2015; Dimidschstein et al., 2016; Mehta et al., 2019). Thus, we sought to identify REs that could show specific activity within CIN subtypes.

### Epigenetic screen for candidate CIN enhancers

We first generated a genome-wide list of pREs likely to be active in immature CINs using an epigenomic approach. We leveraged genetic GFP labeling of immature CINs to purify these neurons before performing ChIP-seq for active (H3K27ac) and repressive (H3K27me3) histone modifications. By performing histone modification ChIP-seq on the non-CIN population in parallel, we were able to identify pREs likely to be not only active in CINs, but also specific to CINs compared with PNs and other cortical cells such as astrocytes. This served as a starting point for identifying cell type-specific pREs. We then combined this with a directed “candidate gene” based approach looking for pREs near genes expressed in CINs. Finally, we integrated our H3K27ac histone ChIP-seq data with published epigenomic datasets from embryonic (TF ChIP-seq) and adult (ATAC-seq) CINs to increase the likelihood that these candidate pREs would be active across CIN developmental stages.

Other groups have taken a similar approach using mainly chromatin accessibility data from ATAC-seq experiments to identify REs for use in AAV targeting CINs (Hrvatin et al., 2019; Mich et al., 2020; Vormstein-Schneider et al., 2020). Candidate regions with enrichment of H3K27ac are likely to give a better success rate than accessible regions determined by ATAC-seq alone because H3K27ac is a more specific mark of active enhancers. This makes our dataset a valuable resource for others seeking to identify additional CIN-specific REs.

One of the main drawbacks of using a genome-wide screen to identify pREs is that it may identify many false positives, as individual epigenetic markers are not sufficient to prove that a locus will function in the cell type under investigation (Nord et al., 2015; Hrvatin et al., 2019; Mehta et al., 2019; Mich et al., 2020; Vormstein-Schneider et al., 2020). However, we postulated that by integrating multiple sources of data we could increase our confidence in the candidate pREs. Thus, we combined our H3K27Ac screen with previously published DLX2, LHX6, and NKX2-1 TF ChIP-seq (Sandberg et al., 2016; Lindtner et al., 2019) to help identify enhancers active in developing CINs. This, however, still does not ensure activity in adult CINs in the absence of further testing.

In addition, H3K27ac enrichment in the CIN population compared with the non-CIN population does not guarantee complete specificity of RE activity to CINs. The non-CIN population is highly heterogeneous, thus a pRE active in CINs and a minority non-CIN cell type, e.g., a subtype of PN, may not show significant enrichment in the non-CIN bulk sample. Any screen will inevitably produce false negatives as well. Sample quality and sequencing depth may affect how many false negatives are produced.

Another consideration is the fact that genomic context can affect expression; even if endogenously active REs are accurately identified, their activity could be altered in heterologous systems such as extrachromosomal AAV vectors. A recent study compared REs in AAVs and transgenic mouse lines to drive expression in glutamatergic cells of the entorhinal cortex and found that only half of the tested AAVs targeted cells in a similar pattern to the corresponding transgenic line (Nair et al., 2020). Despite improvements in computational RE prediction methods, this is likely to mean that individual candidates will continue to need empirical testing. An elegant, recently published method addresses the testing bottleneck by combining massively parallel reporter assays with single-nucleus RNA-seq to test hundreds of candidate enhancer AAVs in a single animal (Hrvatin et al., 2019). They found that a small fraction of tested sequences drove SST^+^ CIN-specific expression as predicted from chromatin accessibility signatures.

Finally, because we used the entire neonatal CIN population (purified from *Gad67-GFP* mice) for H3K27ac ChIP-seq, our screen may not efficiently identify REs whose activity is specific to individual adult CIN subtypes. Our approach can be extended to improve on this by using, for example, sorted CINs from *SST-Cre*, *PV-Cre*, and *VIP-Cre* transgenic animals as has been done for ATAC-Seq (Mo et al., 2015). The major hurdle to this is obtaining sufficient numbers of cells for histone modification ChIP-Seq, which generally requires at least ∼100,000 cells. However, new methods such as CUT&RUN and CUT&TAG are improving the ability to perform epigenetic analyses with cell numbers in the 100–1000 s (Skene and Henikoff, 2017; Skene et al., 2018; Kaya-Okur et al., 2019).

### Discovery of two new CIN-specific REs

We have shown that our two selected candidate pREs, *Arl4d* and *Dlgap1*, can drive robust expression when placed in AAV vectors. The strength of reporter expression and specificity to the GABAergic population are comparable to AAV using the well-characterized *I12b* RE.

Our slice physiology experiments demonstrated that *Arl4d* and *I12b* pRE-driven EYFP is predominantly expressed in fast-spiking CINs. In addition, we have shown that AAV-Arl4d-ChR2-EYFP can rescue behavioral deficits linked to reduced fast-spiking activity in *Dlx5/6*^+/−^ mutants through optogenetic stimulation. Thus, these RE AAVs can drive channelrhodopsin expression at sufficient levels for functional/behavioral studies, which can sometimes be a problem with AAVs targeting specific cell types (Sohal et al., 2009). This demonstrates the wide-ranging utility of our RE AAVs for the neuroscience community.

We also showed that AAV-Dlgap1-ChR2-EYFP-targeted cells were more likely to exhibit regular-spiking than fast-spiking properties in slice physiology experiments. This suggests that specificity for different physiologically defined CIN types might be achievable by selecting different REs. In particular, *Dlgap1_RE* and *Arl4d_RE* seem to differentially label fast-spiking versus regular-spiking neurons. However, when we assessed this further by immunohistochemistry, we found that all three enhancers preferentially labeled PV-expressing neurons. Thus, additional work will be necessary to parse out the differences in specificity reported using electrophysiological and immunohistological analyses.

One possible explanation for the difference between the electrophysiology results and immunostaining for AAV-Dlgap1-ChR2-EYFP is that some cells could express EYFP more strongly than others. These would likely be the cells chosen for patching. In contrast, immunohistochemistry used antibody labeling for EYFP, which could amplify labeling in weakly expressing cells. Thus, immunohistochemistry may sample a broader population of transduced neurons than electrophysiology experiments that depend on live fluorescence. Along these lines, Mehta et al. (2019) found that the *h12R* enhancer drove expression at high and low levels with a bimodal distribution when they assessed reporter expression by *in situ* hybridization. In addition, fast-spiking properties and PV expression, although highly correlated, do not show a 1:1 relationship (Moore and Wehr, 2013). For example, PV-negative fast-spiking Chandelier cells have been reported in the mPFC (Taniguchi et al., 2013), and PV^+^ CINs with non-fast-spiking properties have also been reported (Blatow et al., 2003). One possibility is that AAV-Dlgap1-ChR2-EYFP may preferentially label regular-spiking PV^+^ cells. Of course, our electrophysiology dataset has a smaller sample size than our immunohistochemistry dataset. As such, the discordance between these two methods may simply represent statistical anomalies in our electrophysiological dataset.

### Future utility of CIN-specific AAVs

AAVs with CIN-specific conserved REs offer a significant advantage over the existing mouse transgenic lines because of the ease of use of these tools across species, including in humans and non-human primates (Dimidschstein et al., 2016; Mehta et al., 2019; Mich et al., 2020; Vormstein-Schneider et al., 2020). In addition, RE AAVs injected in small volumes would facilitate effector protein expression and/or gene knock-out in restricted brain regions, and therefore could be used to uncover the physiology and function of CINs in different cortical regions.

REs could potentially be further engineered by mutation or addition of TF binding sites to increase activity or specificity. The REs described in the present study could also be tested in the context of different AAV serotypes, as the specificity of AAV-driven expression can be affected by the serotype and titer of the virus used (Nathanson et al., 2009b). Overall, we have identified novel CIN specific REs and our study describes methodology for identifying potentially hundreds of additional enhancers that could drive CIN subtype-specific expression in AAV vectors.

## Synthesis

Reviewing Editor: Karl Herrup, University of Pittsburgh School of Medicine Decisions are customarily a result of the Reviewing Editor and the peer reviewers coming together and discussing their recommendations until a consensus is reached. When revisions are invited, a fact-based synthesis statement explaining their decision and outlining what is needed to prepare a revision will be listed below. The following reviewer(s) agreed to reveal their identity: Danny Leung.

The manuscript addresses the question of the role of cortical interneuron (CIN) dysfunction in neuropsychiatric conditions such as epilepsy, schizophrenia and autism. In approaching this topic the authors set as their goal the development of a new and more powerful tool that uses viral vectors to discover gene regulatory enhancer elements (REs) that are operative in CIN and other specific cell types. The work uncovered two potential REs, which are compared to the better established Dlx I12b enhancer. Both REs drove CIN-specific reporter expression in adult mice and proved useful in specific experimental settings.

The reviewers of the work both felt that this was a well-constructed study that applies basic epigenomic analysis to improve the technology used to drive CIN specific gene expression. They found the emphasis on the genome-wide screen for putative REs to be interesting and potentially valuable. Minor concerns were expressed that the REs defined here were not all that different in function from published constructs. Technical issues were also raised, in particular the adequacy of the descriptions of techniques offered in the Methods as well as the specific approaches to the bioinformatics. On balance, however, there was considerable enthusiasm for working with the authors to improve the work and see it published in eNeuro.

With regard to the bioinformatics, more details must be provided in the Methods to inform the reader as to how the material was generated. For example, in Figure 2, it is implicit but not spelled out that the authors have re-processed the TF and ATAC data with methods similar to the ChIP data that were generated. Further, it is not evident that there is ATAC-seq signal at the highlighted region as mentioned. Beyond the need for more methodological details, concerns were raised about the peak calling. Two replicates were performed, which would usually mean an irreproducible discovery rate (favored by groups such as the ENCODE consortia) would be determined. With more replicates, it would have been possible to use a package such as diffBind and statistical testing provided from DESeq2/edgeR to establish peaks of low variability, Instead, the authors used MACS2 for differential peak calling. This is acceptable given the q<0.01 used for final peaks. However, there needs to be considerably more information provided in the Methods to allow the reader to assess the results. For example, it is unclear if they have called bdgdiff for each replicate using the 4 bedgraphs: t1 (CIN) t2 (nonCIN) c1 input for CIN and c2 input for nonCIN, essential in making the decision to call peaks as high confidence.

Also, it would be better if there were validation experiments performed for the ChIP-seq datasets. An independent ChIP-qPCR at some of the loci shown in the screenshots in Figure 1 would serve this purpose. Moreover, it would allow for quantitative comparison of enrichment like those mentioned in Figure 2B. The authors used GREAT to assign the associations between an RE and a gene. Although it is a standard for GO analysis of non-coding sequences, GREAT largely only considers linear distance between 2 loci. It would help if, in addition, the authors were to compare definitions with existing lists of enhancer-promoter pairs. For instance, the ENCODE project has an extensive list from various different tissues and cell types. Along the same lines, when asserting that an RE “could regulate Dhx8 in addition to or instead of, Arl4d", incorporation of existing definitions and referring to published higher-order chromatin interactions such as those defined by Hi-C data would help.

The authors aimed to define CIN-specific REs by doing ChIP-seq in sorted cells. This is a sound approach but the non-CIN cells are likely far more heterogenous, making it difficult to ensure that the signal is not simply diluted and that the RE is actually not CIN-specific. Flow cytometry analysis to determine the percentage of co-occurrence of EYFP+ and other cell type markers could address this point and help quantify the specificity of the RE in their in vivo assay. While they would strengthen the manuscript, if these experiments are not possible, it will be essential to point out this limitation in the discussion.

For the RE list that incorporates K27ac, TF ChIP-seq and ATAC-seq datasets, a motif enrichment analysis would be informative. It would also be useful to provide the genomic annotations for CIN-peaks compared to expected distributions (fold-elevated). The authors could analyze whether the identified peaks were more likely to be in intergenic regions or proximal genes in dense regions. Consideration should also be given to comparing the REs they have defined to those found in other mammals (primates/humans/etc) since their motivation is cross-species tools to target CINs specifically. They might also wish to consider running a de novo motif analysis (MEME/DREAM suite or something similar) on the CIN specific, non-CIN, and joint peaks to strengthen the link to specific TFs.

Minor Comments:

1. On line 11, histone ChIP-seq should be histone modification ChIP-seq

2. Line 135 mentions reverse crosslinked chromatin but the protocol was a native ChIP.

3. On Line 446, H3K27ac+ should be H3K27 enriched, as the signal is not binary.

4. Clarify for the reader the meaning of the data showing the enrichment of H3K27ac over gene body and proximal sequence. This is a less standard approach to the questions posed and has no widely accepted meaning in the field; it would help if this line of reasoning was expanded.

5. Line 471 should probably read RE and not GE.

6. Genome browser screenshots lack y-axis values.

7. It would be helpful to include a track on the genome browser screenshots that show the peak definitions.

8. Figure 1A. It is unclear in the cartoon as to what “conservation” applies to. Evolutionary? Sequences only for CINs?

9. Figure 4A. The subtype markers used were not explicitly specified. Please clarify this point. It would also be helpful to see the enrichment of K27ac at the promoters of marker genes.

10. Figure 5. An additional negative (mock AAV) or positive (AAV-I12b-ChR2-EYFP) control would go a long way towards supporting this experiment. The authors should address the question of whether the elements they identify in this paradigm perform similarly to Dlx-I12b (given levels of staining/infection)
