# Supplementary figures and images for: Regulatory Elements Inserted into AAVs Confer Preferential Activity in Cortical Interneurons
Source: eNeuro. 2020 Dec 10;7(6):ENEURO.0211-20.2020. doi: 10.1523/ENEURO.0211-20.2020 (PMC7768279; doi:10.1523/ENEURO.0211-20.2020)

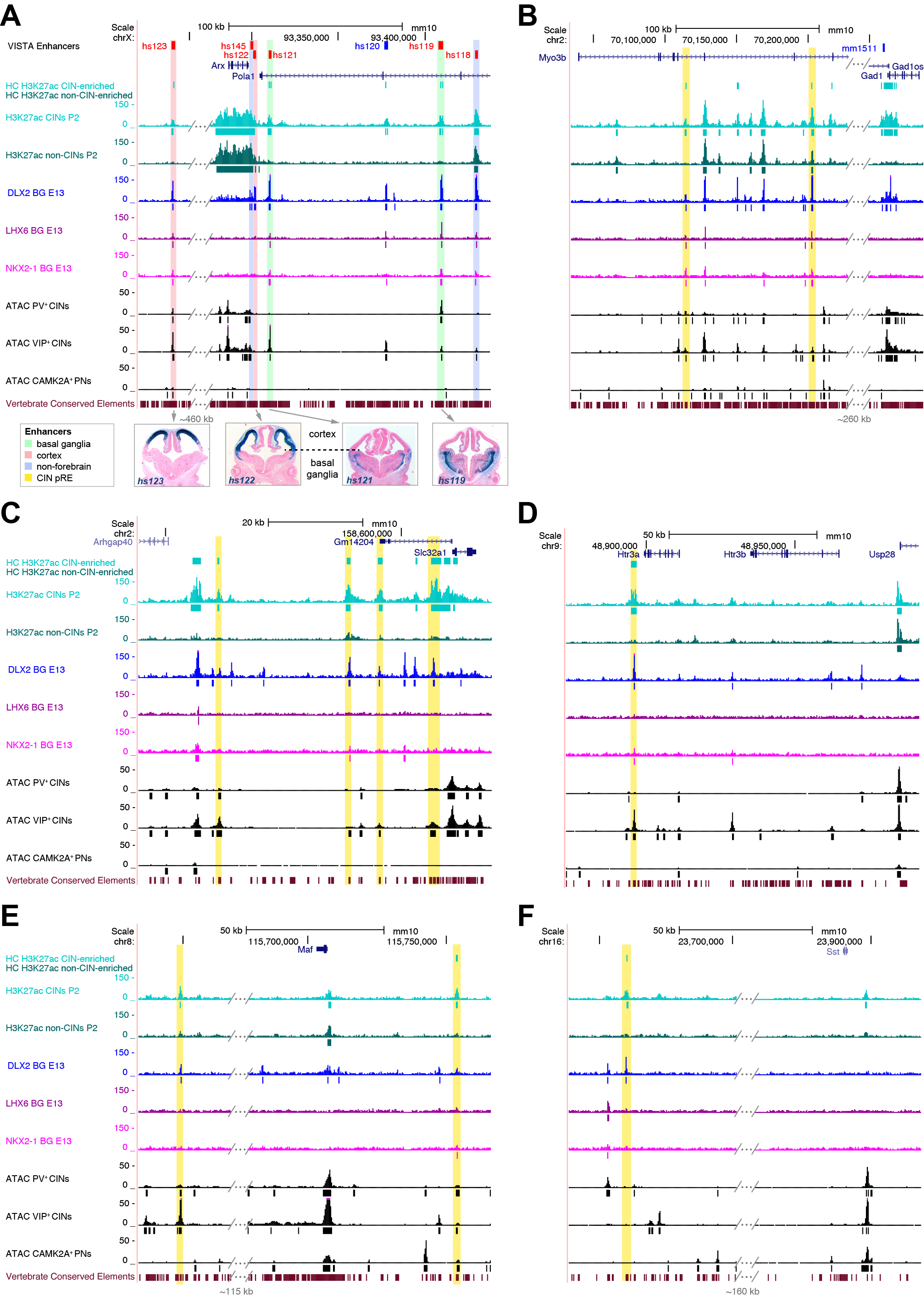

Supplement: Extended Data Figure 3-1 — Epigenetic signatures of additional candidate CIN pREs. A, The Arx locus contains several validated enhancers. Enhancers with activity in the basal ganglia (green highlights) have HC H3K27ac CIN-specific peaks, DLX2, LHX6, and NKX2-1 TF binding peaks, and enhanced accessibility by ATAC-seq in P30 PV+ and/or VIP+ CINs but not in CAMK2A+ PNs. Enhancers active in the cortex (red highlights) or outside the telencephalon (blue highlights) lack this particular signature: hs123 is also accessibly by ATAC in CAMK2A+ PNs; hs122 has H3K27ac enrichment in both CINs and non-CINs. Sections showing transient transgenic enhancer activity at E11.5 are taken from the VISTA Enhancer Browser (https://enhancer.lbl.gov; Visel et al., 2013). B–F, Epigenetic signatures of CIN-specific pREs (yellow highlights) near the CIN genes Gad1, Slc32a1 (Vgat), Htr3a, Maf, and Sst. Download Figure 3-1, TIF file. [file enu-eN-MNT-0211-20-s04.tif]

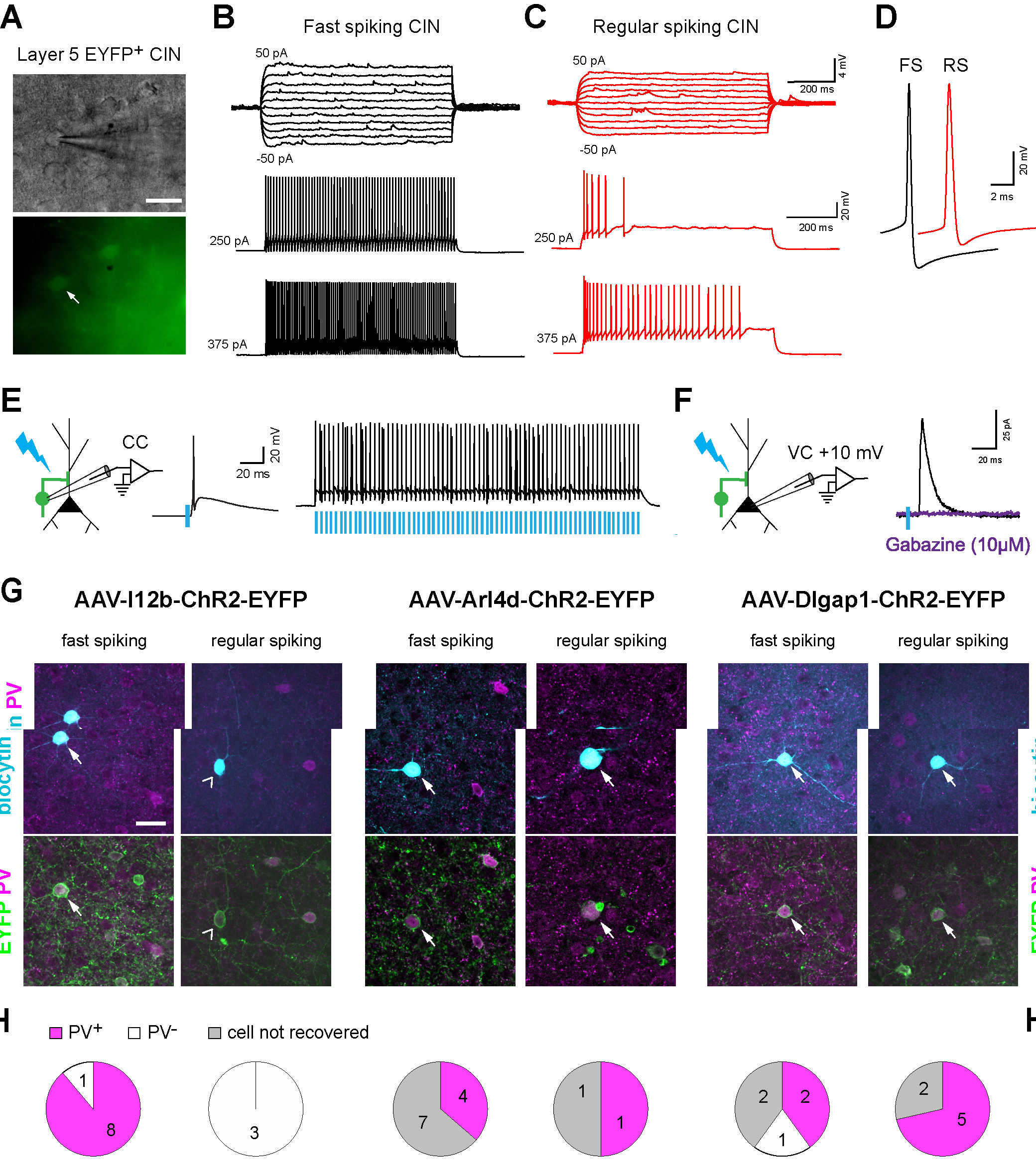

Supplement: Extended Data Figure 4-1 — Electrophysiological characterization of layer 5 CINs targeted by RE-AAVs. A, DIC and fluorescent images showing that EYFP+ layer 5 CINs in mPFC were targeted during patch clamp recordings. Scale bar: 25 µm. B, Example voltage traces in response to subthreshold current injections (–50 to +50 pA, top) and to suprathreshold current injections (250 and 375 pA) in an EYFP+ fast-spiking (FS) CIN. C, Same as B for an EYFP+ regular-spiking (RS) CIN. D, Example voltage traces showing single APs recorded from EYFP+ FS and RS CINs. E, left, Recording configuration showing that current clamp recordings were obtained from layer 5 EYFP+ CINs. Right, APs were reliably elicited in EYFP+ CINs in response to blue light (470 nm) flashes during patch clamp recordings. F, left, Recording configuration showing that voltage clamp recordings (+10 mV) were obtained from EYFP-negative layer 5 PNs. IPSCs (black trace) were recorded in PNs in response to blue light flashes. Currents were completely blocked by application of gabazine (10 µm). G, Representative images of post hoc labeling for biocytin, PV, and EYFP in recorded cells for each enhancer AAV injection. Arrows indicate PV+ recorded cells and open arrowhead indicates a PV– recorded cell. Scale bar: 25 µm. H, Pie charts showing the percentages of biocytin-labeled FS and RS CINs expressing PV by post hoc labeling. Some cells were not recovered after patching. Number of cells of each type is indicated. Download Figure 4-1, tif file. [file enu-eN-MNT-0211-20-s06.tif]
